# Mapping the Influence of Infant–Parent Relational Quality on Life Course Relationships: A Scoping Review of Prospective Cohort Studies

**DOI:** 10.1007/s10567-025-00527-5

**Published:** 2025-06-08

**Authors:** Felicity Painter, Jacquelyn Harverson, Gabriella King, Tracy Evans-Whipp, Melissa J. Green, Kayla Mansour, Lu Zhang, Sarah Whittle, Daniel Liontos, Craig A. Olsson, Jennifer McIntosh, the Australian Early Relational Health Network

**Affiliations:** 1 The Bouverie Centre, La Trobe University, 8 Gardiner Street, Brunswick, VIC 3056 Australia; 2 Centre for Youth Mental Health, University of Melbourne, Parkville, VIC Australia; 3 Orygen, Parkville, VIC Australia; 4 SEED Lifespan Strategic Research Centre, School of Psychology, Faculty of Health, Deakin University, Burwood, VIC Australia; 5 Centre for Adolescent Health, Murdoch Children’s Research Institute, Melbourne Royal Children’s Hospital, Parkville, VIC Australia; 6 Department of Paediatrics, The University of Melbourne, Melbourne Royal Children’s Hospital, Parkville, VIC Australia; 7 Discipline of Psychiatry and Mental Health, University of New South Wales, Sydney, NSW Australia

**Keywords:** Relational health, Attachment, Caregiving, Parent–child dyad, Cohort studies

## Abstract

This review is part of a series of scoping reviews of population-based cohort studies, designed to inform public health approaches to strengthening the relational ecology of early child development. Here, we scoped prospective cohort studies that have assessed the association between infant–parent relational health during the first years of life (from conception to age three years) and relationship quality across the life course. Studies were included if they explored predictor measurement of infant relational behaviors with their parent (e.g., attachment status) or parent relational behaviors with their infant (e.g., parental sensitivity), and a relational health outcome across the whole social ecology. A systematic search of the literature was conducted in September 2023. The search identified 15,454 articles, of which 108 studies were deemed eligible for inclusion. An additional nine articles were identified via citation searching resulting in 117 included studies. We examined associations across four developmental periods (early childhood, middle childhood, adolescence, and adulthood), in a range of affiliative relationships, disaggregating outcomes in intra-familial and extra-familial relationships. Despite considerable variation in measurement and sampling, we found consistent evidence of enduring influence of infant–parent relationship quality for relational functioning well into adolescence. For some relationship forms, this extended into adulthood. Understanding the reach of early parent–infant relational quality onto life course relational outcomes has potential to inform public health policy settings.

**Supplementary Information:**

The online version contains supplementary material available at 10.1007/s10567-025-00527-5.

## Introduction

Relational health has long been understood as central to overall health and wellbeing across the life course. The importance of *early* relational health is now emerging as a priority focus area for investment in public health and health services, globally (Paul Ramsay Foundation, 2022; Willis et al., 2024). This focus on early relational health is supported by multiple developmental theories, encompassing diverse constructs including attachment, trust, bonding, sensitivity, and emotional connection. Each of which ultimately converge in their shared objective of describing unique qualities of the main carer–child relationship (Dumitriu et al., 2023). Developmental theories from Bowlby and Erikson onward align in their shared focus on trust in others as a cornerstone of early relational health (Markson & Luo, 2020), with expectations of trust thought to generalize during childhood and later life, across distributive affiliative systems (kin and non-kinship relationships) over time (Bowlby, 1979; Bowlby et al., 1992; Erikson, 1994). As such, infancy and early childhood represent key developmental periods during which fundamental relational ecologies begin to form, while at the same time representing key timeframes for prevention and early intervention.

Attachment theory provides one of the most comprehensive frameworks for understanding early interactional experiences within the caregiver–child dyad, and the correspondent influence in shaping relationships over time. Early theory about the origins of first relationship security (Ainsworth, 1985; Solomon & George, 1996) is now well supported by meta-analytic research (e.g., Madigan et al., 2024). A child’s attachment status is significantly shaped by their parent’s ability to recognize and accurately interpret attachment-based signals and to provide a sensitive and timely response that appropriately meets the needs of their child. Children who experience coherent, predictable, and consistent care tend to develop secure attachment relationships (De Wolff & Van Ijzendoorn, 1997; O’Neill et al., 2021), which are associated with positive perceptions of, and trust in, their caregiver. In contrast, insensitive or relatively unresponsive caregiving tends to be associated with the genesis and maintenance of attachment insecurity (McIntosh et al., 2024). These early interactional patterns become internalized by the developing child, and by the time of entry to pre-school, result in the formation of internal working models of self and others in the social world (i.e., perception of self and others; Li et al., 2021). These models guide expectations of and responses to others and scaffold subsequent interactions within close relationships.

The extent to which early relational health within the main carer–child dyad continues to shape expectations about a range of future relationships across early childhood and into adolescence has been examined in the context of both attachment (e.g., Deneault et al., 2021; Groh et al., 2017; Opie et al., 2021; Pallini et al., 2014) and parent-initiated relational quality (e.g., sensitivity; Rodrigues et al., 2021). While examination of these associations is well documented up to adolescence, beyond this, evidence is disparate, less numerate, and largely un-harvested. Further, no review to date has collated prospective research focused on the reach of early relational health quality within the main carer–child dyad to life course relational outcomes, including and beyond the child–parent relationship. This limited knowledge is a constraint to social policy development around when, and for how long, public health investments need to be targeted and maintained to protect, nurture, and strengthen life course relational health.

To address this gap in knowledge, we conducted a systematic scoping review to synthesize the findings of cohort studies that map longer-term relational outcomes, from conception to three years, into childhood, adolescence, and adult life.

Specifically, we aimed to:

- scope prospective population-based cohort studies that have assessed the association between early main carer–child relational health during the first years of life (from conception to age three) and relationship quality across the life course,
- identify and examine this pathway across four developmental periods (early childhood, middle childhood, adolescence, and adulthood), across a range of affiliative relationships, disaggregating intra-familial and extra-familial relationships, for both specified (e.g., intimate partner) and generalized (e.g., adult attachment status) bonds, and
- describe and synthesize the key findings.

Our review forms part of a broader series of systematic scoping reviews designed to describe literature from population-based cohort studies, and community (public health) trials, to inform public health approaches to promoting early relational health in populations.

## Methods

A scoping review was the preferred methodology for our policy-driven, facilitating high-level mapping of longitudinal research conducted in our area of interest. Unlike a systematic review or a meta-analytic process, it accommodated variability in conceptualization of early relational health in the main carer–child dyad from conception to age three, and in the operationalization of findings in multiple relationship forms, and across all developmental periods.

The scoping review was guided by the Joanna Briggs Institute (JBI) approach to scoping reviews (Peters et al., 2020) to understand the extent of knowledge and identify the key knowledge gaps. We extend on this methodology to further include a narrative synthesis of the study findings. Reporting utilized the Preferred Reporting Items for Systematic Reviews and Meta-analyses Extension for Scoping Reviews (PRISMA-ScR; Tricco et al., 2018). The PRISMA-ScR Checklist is provided in the Supplementary Information (see Online Resource 1).

### Eligibility Criteria

Eligibility criteria were developed in line with the JBI recommended Population-Concept-Context (PCC) framework (Pollock et al., 2023).

#### Population

Studies of general population, non-clinical cohorts, followed prospectively were eligible for inclusion. We considered samples with baseline measurement of main carer–child dyadic relationship quality taken within the conception to offspring age three range and subsequent outcome measurement of relationship quality between child and others from age 4 years onward. No limitations were placed on main carer age at predictor measurement. Child age at outcome had no upper limit.

#### Concept

We considered studies reporting on the longitudinal association between early relational health within the main carer–child dyad and subsequent relationship quality between child and others across the life course. Studies were eligible for inclusion if the predictor measure reflected child- or parent-behavioral initiatives contributing to dyadic relational health. While inextricably linked, these constructs are importantly distinct from one another in measurement. Their delineation offers precision in research- and policy-related recommendations arising from the current review.

- Child behavioral initiatives contributing to dyadic relational health with their parent (age 0–3 years) were defined as relationally oriented interaction with a main caregiver by the child, for example, attachment status,
- Parent behavioral initiatives contributing to dyadic relational health with their child (from conception to age three years) were defined as relationally oriented interaction with the child by a main caregiver, for example, caregiving sensitivity, parent emotional availability, parenting strategies, and parenting practices (both positive and maladaptive),

Outcomes of interest spanned the interpersonal social ecology (Bronfenbrenner, 2005), including:

- Immediate and extended family relationships, peer and school relationships (e.g., teacher-reported social functioning), intimate–partner relationships, and workplace relationships,
- General representations of relational experiences such as non-specified attachment (i.e., relationships that are not measured in the context of a relationship with a specific person/s (e.g., Adult Attachment Interview [AAI]; George et al., 1985)).

No limitations were placed on predictor or outcome measure type (e.g., observational, parent-reported). However, a condition for inclusion was the report of a self-other referent measure or a relevant subscale comprised of predominantly self-other referent items. That is, scale items had to examine the target individual in relation or reference to another person/s (e.g., the item *If you wanted to join a group of kids playing and they said no, how would you feel?* on the Children’s Rejection Sensitivity Questionnaire [CRSQ]; Downey et al., 1998). Other measures clearly present a shade of gray. For example, generalized parent-reported externalizing behaviors were considered too broad and ineligible for inclusion, unless reported on at the subscale level (i.e., Aggressive Behavior subscale of the Child Behavior Checklist [CBCL]; Achenbach & Edelbrock, 1991), or in the context of teacher reports where self-other referent items are primarily focused on a specified relationship with peers.

#### Context

Intervention studies were excluded. Clinically indicated samples were excluded.

### Information Sources

The search was first conducted in September 2023. Three electronic databases were searched for relevant articles: MEDLINE (EBSCOhost), PsycINFO (EBSCOhost), and Embase (Ovid). Snowball searching of pertinent articles was also carried out to identify additional articles.

### Search

The search strategy combined three core concepts. Concept 1 was specifically designed for use across all reviews within the current series and as such broadly encompassed terms pertaining to the child and family relational ecology. This meant, the identification of articles reporting on the relational correspondence from the main carer-infant relationship only was a parameter defined by the eligibility criteria specific to this review. Concept 2 defined the predictor time period (conception to age three years), and Concept 3 defined the relevant study design (prospective, longitudinal). Due to the intentionally broad scoping nature and anticipated variability in potential outcomes of interest, outcome terms were not specified in the search strategy. The full electronic search strategy is provided in Supplementary Information (see Online Resource 2).

Studies were limited to peer-reviewed articles, published in the English language. No date limitations were applied.

### Selection of Sources of Evidence

All records were imported into the SEED LitQuest tool for screening and duplicates were removed. At the title and abstract level, double screening was carried out on 15% of all articles, following which an inter-rater check was performed on 100 articles to assess the feasibility of moving to a single screening process. Inter-rater reliability between screeners was > 95% and as such, articles were single screened until a stop-point indicated by the LitQuest. At the full text level, all articles were double screened. The screening process was carried out by three independent researchers (FP, JH, GK), with discrepancies resolved via discussion to reach consensus.

### Data Extraction

Included studies were tabulated into a standardized data extraction form detailing study information, predictor and outcome variables, measure and relationship type, and key findings. For each study, eligible predictor and outcome variables have only been extracted when a relevant analysis was also reported. See Online Resource 3 for data extraction and reference list of all included studies.

### Establishing the Living Review

As outlined by Cochrane Collaboration guidelines for the production and publication of reviews in living mode (Brooker et al., 2019), we plan to monitor the emergence of new literature in this field through periodic updates of the current review. This process will involve replicating the initial search across each of the relevant databases, importing newly identified articles into LitQuest for screening against the established eligibility criteria, and extracting data from new studies to integrate with the existing synthesis. The review will be updated iteratively over time, with regular assessment to determine whether it remains feasible and necessary to continue in a living review mode.

## Results

### Selection of Sources of Evidence

**Fig. 1:**
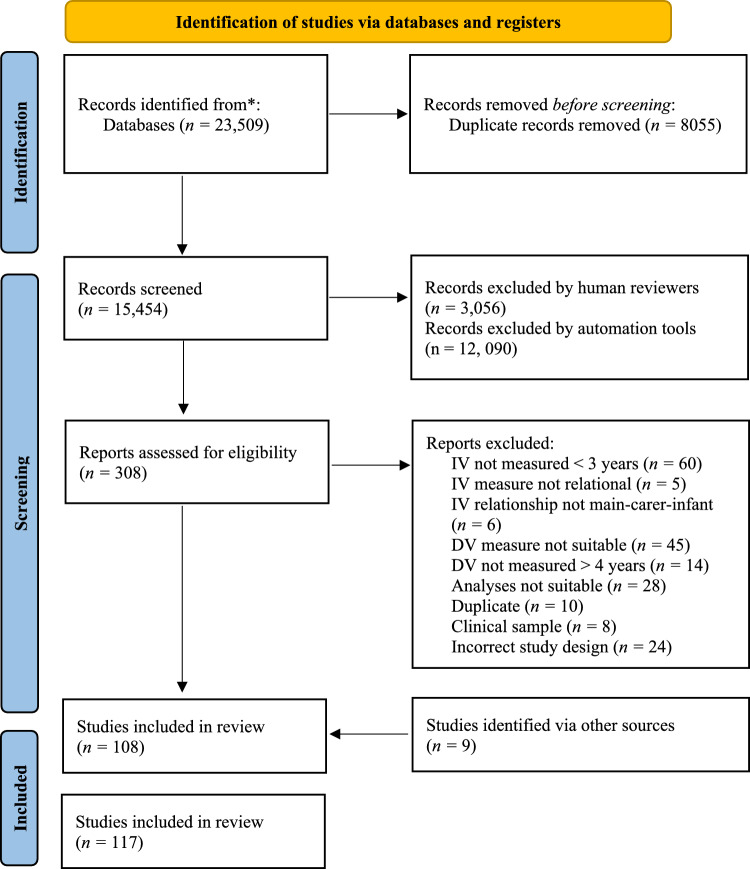
PRISMA flow diagram of the study selection process

The search returned 15,454 articles excluding duplicates. At the title and abstract level, 3364 articles (21.8%) were manually screened, with 3056 of these excluded. The remaining 12,090 articles were excluded by LitQuest. Full-text screening was carried out on 308 articles. A total of 108 articles were eligible for inclusion. Nine additional articles were identified via citation searching. The findings below are therefore drawn from extracted data of 117 studies conducted with longitudinal cohort samples. Screening and eligibility are reflected in the PRISMA flowchart (Page et al., 2021; see Fig. 1).

### Characteristics of Sources of Evidence

Articles were published between 1985 and 2022. Sixty-two percent of studies reported on participant samples in the United States, 10% of studies were from Germany, and 5% were from Canada. The remaining studies (23%) were conducted across 11 countries globally (see Online Resource 3). Sample size varied significantly across studies, ranging from 28 to 6850 parent–child dyads. Within the 117 studies identified, 63 unique cohorts were represented. Noting 20 studies (17%) did not report or specify the cohort from which data were derived.

### Results of Individual Sources of Evidence

#### Predictors

Predictor measures classified under the child’s initiative included attachment, child emotional availability to parent, affection to parent, responsiveness and involvement, gaze, connected behavior, compliance, avoidance, and reciprocity. Predictors classified under the parent initiative included measures of caregiving sensitivity, parent emotional availability, parenting strategies and practices, engagement, parental separation anxiety, co-regulatory behaviors, bonding, supportive behaviors, and communication.

The 117 included studies reported on predictor variables operating in the developmental period from 3 months to 3 years. Noting we found no prospective cohort research examining the parent relationship during the pregnancy period as a predictor. The child’s relational initiative with the parent only, was reported by 55 studies (47%). Of these, the majority (*k* = 49; 89%) specifically measured attachment observationally with the Strange Situation Procedure (SSP; Ainsworth et al., 1978) or by the Attachment Q-Set (AQS; Waters, 1995). The parent’s relational initiative with the infant was reported by 40 studies (34%), and 22 studies (18%) examined predictors pertaining to the initiative of both the child and the parent. Predictor assessment of the mother–infant relationship only was reported in 66 studies (56%), the father–infant relationship only in one study, and mixed samples in 50 studies (43%). See Online Resource 3 for full details.

#### Outcomes

Outcomes across four developmental epochs were defined: early childhood, middle childhood, adolescence, and adulthood. Within each epoch, we disaggregated outcomes by eight relationship structures as determined by reported outcomes of included studies: parent–child, sibling, family, peer, child–teacher, romantic, generalized attachment, and general relationship representations*.* While the latter two both pertain to outcomes in a non-specified relational subsystem, generalized attachment (i.e., via the AAI or Attachment Assessment Script [ASA]; George et al., 1985; Waters & Waters, 2006) has been separated to distinguish continuity in attachment across the life course.

Included studies reported on outcomes from 4 to 35 years. Disaggregation of study volume and significance according to both outcome and developmental period are presented in Table 1. To note, in cases where *trajectories* of an outcome are reported/defined, the study is situated and considered only in the latest developmental epoch.

**Table 1:** Main carer-infant relational security and associations with child's later relationship outcomes: study volume and percentage of significant outcomes across developmental stages and relationship forms

Associations with child’s later relationship quality by developmental stage	Relationship type								
	Parent–child *k* (% Sig)	Sibling *k* (% Sig)	Family *k* (% Sig)	Peer *k* (% Sig)	Teacher *k* (% Sig)	Romantic *k* (% Sig)	Generalized attachment *k* (% Sig)	General *k* (% Sig)	Total studies *k* (% Sig)
	*Predictor* = *Infant Attachment Status/ Infant Relational Quality with the Parent, 0–3 years (k* = *73 unique studies)*								
Early Childhood (4–6 yrs)	24 (91.67)	1 (100)	1 (0)	14 (92.86)	2 (50.0)	–	–	3 (100)	42 (90.48)
Middle Childhood (7–11 yrs)	8 (100)	–	1 (100)	10 (70.0)*	–	–	1 (100)	4 (100)	19 (84.21)
Adolescence (12–17 years)	3 (33.33)	–	–	6 (83.33)	–	–	4 (50.0)	2 (100)	13 (76.92)
Adulthood (18 yrs +)	1 (100)	–	–	1 (100)	–	6 (83.33)		5 (60.0)	12 (83.33)
	*Predictor* = *Parent Relational Quality with the Infant, 0–3 years (k* = *61 unique studies)*								
Early Childhood (4–6 yrs)	12 (100)	–	–	17 (76.47)	1 (100)	–	–	7 (71.43)	35 (80.0)
Middle Childhood (7–11 yrs)	4 (75.0)	–	–	13 (84.62)	1 (100)	–	–	3 (66.67)	20 (85.0
Adolescence (12–17 yrs)	–	1 (100)	–	2 (100)	–	1 (0.00)	1 (100)	5 (80.0)	10 (80.0)
Adulthood (18 yrs +)	–	–	–	2 (100)	–	2 (100)	5 (60.0)	1 (100)	8 (75.0)

*Significant findings within group demonstrate contradictory evidence; Total studies is the total number of individual studies within each age group accounting for duplication across systems; Significance of total unique studies is representative of significance achieved in any relational structure within that specific developmental epoch. The general column quantifies outcomes measured in a non-specified relational subsystem (i.e., relationships not measured in the context of a relationship with a specific person/s). The generalized attachment column quantifies studies reporting on a general indicator of what is termed “attachment state of mind” via the AAI and has been separated from general relationships to distinguish continuity in attachment across the life course

*k* = number of studies; Sig = Evidence of a significant association on any variables examined; yrs = years

### Synthesis of Results

**Fig. 2:**
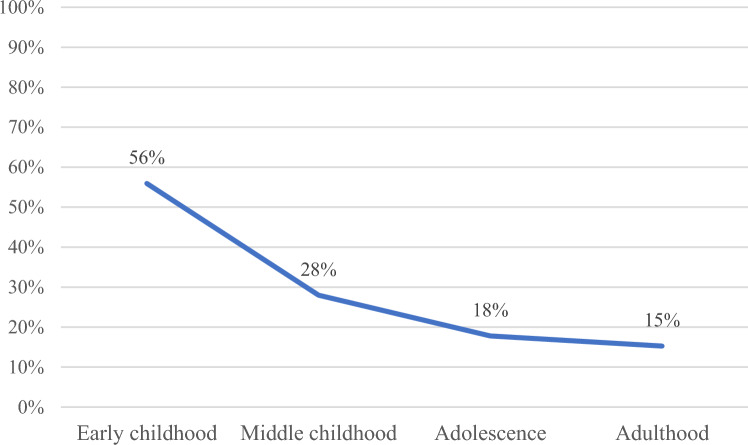
Percentage of studies with outcomes at each developmental epoch (k = 117)

Approximately 60% of studies (*k* = 66) reported relational health outcome/s in the early childhood period, with study volume diminishing across the life course. Figure 2 provides graphical representation of study volume across each developmental epoch. We summarize the findings below in line with this across the eight relational structures. Synthesis of results has been provided where at least three studies fall within a specific developmental subgroup.

#### Early Childhood (4–6 Years)

##### Infant Relational Initiative with Parent at 0–3 Years and Child Relational Outcomes at 4–6 Years

Associations from the child’s behavioral initiative during early childhood were predominantly explored in parent–child and peer relationships. Twenty-four studies (21%) examined links between infant–parent and later child–parent relationship quality. Of these, 19 focused on continuity of attachment with 90% (*k* = 17) providing evidence for continuity between infancy and early childhood for both secure and disorganized attachment status (Bar-Haim et al., 2003; Celia et al., 2018; Cicchetti & Barnett, 1991; Fish, 2004; Gloger-Tippelt et al., 2002; Goffin et al., 2018; Grossmann et al., 2002; Howes & Hamilton, 1992; Howes et al., 2011; Jacobsen et al., 1997, 2000; Levendosky et al., 2011; Lounds et al., 2005; Main & Cassidy, 1988; Main et al., 1985; Meins et al., 2018; Wartner et al., 1994). Two studies (Borghini et al., 2018; Trapolini et al., 2010) did not find links from attachment in infancy to early childhood. In each, the outcome measure was the Attachment Story Completion Task (ASCT; Green et al., 2000), a story‐stem technique, using dolls and props to begin stories with attachment themes that the child then completes. In contrast, the 16 studies that noted significant associations all involved coding of observed interactional behavior between child and parent. Remaining studies (*k* = 5; 4.3%) examining the parent–child relationship as an outcome demonstrated significant associations from predictors of infant–parent relational health and children’s emotional availability (Barry & Kochanska, 2010; Celia et al., 2018; Harris et al., 2021), as well as negative relational dimensions (e.g., separation anxiety, child negativity; Dallaire & Weinraub, 2005; Rispoli et al., 2013).

Moving to the peer relationship, we note considerable variability in outcome measurement. Despite this, findings consistently demonstrate significant links between infant–parent attachment security and positive behaviors in early childhood (e.g., competence, pro-sociality). Equally, the association is consistent for early insecure attachment and negative peer-related outcomes (e.g., aggression, externalizing behaviors [teacher-report]). Children’s general relational functioning with others during childhood was also reported in three studies (2.6%). Findings of relevant studies show significant links from infant attachment insecurity to subsequent externalizing behaviors, namely, social problems (Hubbs-Tait et al. 1994), disruptive behavior (Kochanska et al., 2008), and aggression (Burgess et al., 2003).

##### Parent Relational Initiative with Infant at 0–3 Years and Child Relational Outcomes at 4–6 Years

Parent initiated relational predictor measures linked to child outcomes at 4 to 6 years were of emotional availability, maternal sensitivity, or responsiveness. All predictor measures were observational (e.g., SSP; Ainsworth et al., 1978, Observational Record of the Caregiving Environment; NICHD Early Child Care Research Network, 1996) with one study also reporting on parent-reported separation anxiety (i.e., Separation Anxiety Scale; Hock et al., 1989).

As with the predictors of the infant’s relational initiative, we find significant direct effects of relational predictors of the parent’s initiative on the quality of the parent–child relationship, across all examined outcome measures (i.e., emotional availability, separation anxiety, co-regulation with parent) in early childhood. Mediating effects of attachment quality in these associations are also reported (Licata et al., 2015).

Associations between parent-initiated relational quality and children’s later functioning in peer structures during early childhood were examined in 17 studies (14.5%). Positive outcomes of interest included pro-social behaviors, social competence, and friendships, with significant associations in the expected direction in 85% of relevant studies (Blandon et al., 2010; Degnan et al., 2015; Ensor & Hughes, 2009; Heuser et al., 2018; Liu et al., 2009; Raikes & Thompson, 2008; Reyes et al., 2019; Takahashi et al., 2015; Wang et al., 2016; Zhang, 2012). Similarly, negative outcomes including aggression and problem behaviors were significant in 75% of relevant studies (Blandon et al., 2010; Raikes & Thompson, 2008; Rubin et al., 2002; Smeekens et al., 2007; Vitaro et al., 2006; Wang et al., 2016). No cohort research was found examining links between parent–infant relational quality and subsequent sibling, family, or teacher–child relationship functioning during early childhood.

General representations of relational functioning during early childhood as predicted by the parent’s behavioral initiative with their child fell into two main categories: parent-reported aggression and general social competence. While studies demonstrated significant associations varying in strength across both categories (i.e., correlative evidence, direct and indirect effects, interactional), all were in the expected direction (Boutwell et al., 2012; Ettekal et al., 2020; Kochanska et al., 2008; Lee et al., 2013; Mills-Koonce et al., 2022; Rispoli et al., 2013; Van Ryzin et al., 2015).

#### Middle Childhood (7–11 Years)

##### Infant Relational Initiative with Parent at 0–3 Years and Child Relational Outcomes at 7–11 Years

Studies examining links to child–parent relational outcomes during middle childhood (*k* = 8; 6.8%) were all specifically focused on attachment status measured via observation (SSP and/or AQS; Ainsworth et al., 1978; Waters, 1995). All studies provide evidence for continuity of attachment across each of these developmental epochs, starting at 12 months (Grossmann et al., 2002). Some within-construct outcome variation was present. For example, Kochanska et al. (2015) report on significant links to children’s obligation to obey but not in their trust of parents. Evidence specific to these variables was correlative.

As with links to intra-familial outcomes, of 10 studies (11.7%) examining children’s peer relationships during middle childhood (Aviezer et al., 2016; Bosquet & Egeland, 2006; Cao et al., 2021; Carlson et al., 2004; Englund et al., 2011; Lewis-Morrarty et al., 2015; Raikes & Thompson, 2008; Shi et al., 2012; Shulman et al., 1994; Ziv et al., 2004), 90% used infant attachment as the predictor. Specific peer outcomes of interest across studies were conceptually similar (e.g., peer competence, peer liking, friendship); however, findings were mixed. One study (Cao et al., 2021), conducted in China, demonstrated significant effects that were contrary to the expected direction, whereby children’s connectedness with their parent during infancy was negatively associated with peer liking during childhood. The authors note the unexpected direction of findings may reflect the maladaptive impact of child connectedness in the context of recent macro-social and cultural change in China. It is worth noting that the evidence was predominantly correlational across studies and solely correlational in all cases of non-significance. This is important to recognize given Bosquet and Egeland (2006) indicated non-significant correlations from infant attachment insecurity to peer relationship functioning, but significant direct effects in the expected direction according to regression/path analysis.

In associations with general relational functioning during middle childhood, three studies examined infant attachment status as the relevant predictor measure (Boldt et al., 2020; Cyr et al., 2014; Kim et al., 2014), and one focused on infant’s mother-directed gaze (Bedford et al., 2017). While significance was observed between variables of interest for all studies within this group, associations for Bedford et al. (2017) and Kim et al. (2014) were contingent on the interactional influence of parent-related factors (e.g., maternal sensitivity and parental discipline).

##### Parent Relational Initiative with Infant at 0–3 Years and Child Relational Outcomes at 7–11 Years

Four studies (3.4%) examined the influence of relational predictors of the parent initiative (e.g., sensitivity, responsiveness) in the 7–11 age range. Three found significant associations with parent–child attachment status and behavior in middle childhood. Combined and interaction effects of child-focused predictors (i.e., attachment security) were notable here—i.e., parental sensitivity and infant attachment in combination predicted middle childhood child–parent attachment (Grossmann et al., 2002; Miller et al., 2019). As with other developmental epochs, some studies found interactional effects. For example, fathers’ play sensitivity and infant–mother quality of attachment predicted children’s internal working model of attachment at age 10 (Grossmann et al., 2002).

In contrast to research in the early childhood period, the quality and strength of evidence linking early parent-initiated relational quality (both positive and negative) and peer functioning in middle childhood appear greater (*k* = 13), despite variation in peer outcome type. Specifically, 85% of studies demonstrated significant associations in the expected direction (Barker et al., 2008; Cao et al., 2021; de Vries et al., 2018; Galán et al., 2017; Heuser et al., 2018; Ostrov et al., 2022; Pears et al., 2013; Raikes & Thompson, 2008; Reyes et al., 2019; Russell et al., 2016; van den Berg et al., 2017).

Examination of links to general representations of relational functioning during middle childhood provides preliminary evidence for the influence of parenting behaviors on children’s social anxiety and callousness (Lorenzo et al., 2022; Shaw et al., 2012). Findings pertaining to the influence of parenting behaviors on child aggressive behavior approached significance (Ettekal et al., 2020).

#### Adolescence (12–17 Years)

##### Infant Relational Initiative with Parent at 0–3 Years and Adolescent Relational Outcomes at 12–17 Years

Three cohort studies to date have examined prediction from infant attachment to adolescent parent–child relationship quality (Becker-Stoll et al., 2008; Carlson, 1998; Englund et al., 2011). Evidence is, however, weak with only one study reporting significance (Becker-Stoll et al., 2008). As with studies situated in middle childhood, and despite variation in outcome measures used (i.e., observational, teacher-, self-reported), adolescent–peer relational functioning was consistently associated with early child–parent relationship quality (Carlson et al., 2004; Englund et al., 2011; Feldman et al., 2013; Haydon et al., 2012; Vieth et al., 2022). For prediction of peer relationship quality, however (i.e., socially interactive behaviors), no association was observed (Zimmermann et al., 2001).

Continuity of attachment from infancy to adolescent attachment representations as measured by the AAI was explored in four studies (3.4%; Beijersbergen et al., 2012; Grossmann et al., 2002; Hamilton, 2000; Zimmermann et al., 2001). Findings were mixed with significant associations reported by 50%. Continuity of security following infant adoption was predicated on continuous high-level maternal sensitivity, and relative increase in maternal sensitive support from early childhood to adolescence predicted children’s change from insecurity in infancy to security in adolescence (Beijersbergen et al., 2012).

##### Parent Relational Initiative with Infant at 0–3 Years and Adolescent Relational Outcomes at 12–17 Years

No studies looked at parent specific behaviors in infancy and subsequent parent–child relationship during adolescence. Similarly, studies reporting on sibling (Dantchev & Wolke, 2019), peer (Haydon et al., 2012; Ostrov et al., 2022), romantic (Goldberg et al., 2019), and generalized attachment (AAI) relationships (Grossmann et al., 2002) were minimal. Five studies (4.3%) did, however, explore the influence of parenting sensitivity on general representations of relational interactions during adolescence (Galán et al., 2017; Godleski et al., 2019; Lorenzo et al., 2022; Streit & Davis, 2022; van der Voort et al., 2014). These studies predominantly focused on children’s exposure to negative/maladaptive parent behaviors and subsequent negative outcomes (e.g., social anxiety, rejection sensitivity, reactive violence). Four studies (Godleski et al., 2019; Lorenzo et al., 2022; Streit & Davis, 2022; van der Voort et al., 2014) found significant associations in the expected direction, and the remaining study (Galán et al., 2017) reported findings that approached significance on a relevant outcome variable.

#### Adulthood (18–35 Years)

##### Infant Relational Initiative with Parent at 0–3 Years and Relational Outcomes at 18–35 Years

One study specifically reported on continuity of attachment within the parent–child dyad from early childhood (12 months) to adulthood (18 years) reporting significant correlations from attachment to parental derogation but not parental idealization (Roisman et al., 2000). Remaining studies exploring outcomes in adulthood primarily reported on romantic relationship quality (Englund et al., 2011; Girme et al., 2021; Haydon et al., 2012; Raby et al., 2013; Roisman et al., 2005; Salvatore et al., 2011) and adult generalized attachment (Raby et al., 2013; Schoenmaker et al., 2015; Steele et al., 2014; Weinfield et al., 2000, 2004). While quality of the evidence is mixed (i.e., correlational, regression), of the six studies reporting on outcomes pertaining to intimate partner relationships (e.g., romantic attachment, relationship effectiveness, conflict, satisfaction, stability), all bar one (Raby et al., 2013) demonstrated a significant association with children’s early attachment status.

As referenced above, continuity from infant attachment to adult generalized attachment (according to the AAI and ASA) was examined in six studies (5.1%). Importantly, findings within this group were mixed, with recognition for additional life course factors that differentiated continuity and discontinuity of attachment. One study in particular (Weinfield et al., 2000) highlighted the relevance of lawful discontinuity in understanding disruption to attachment patterns over time, wherein exposure to risk factors post-infancy (e.g., prolonged hardship, traumatic loss, and abuse) can disrupt continuity of secure attachment patterns.

##### Parent Relational Initiative with Infant at 0–3 Years and Adult Relational Outcomes at 18–35 Years

Similarly, evidence pertaining to familial relationship quality in young adulthood as predicted by parent’s relational initiative is missing. Studies examining peer (Reyes et al., 2021; Zayas et al., 2010) and romantic (Haydon et al., 2012; Zayas et al., 2010) relationships as a function of early parent–child interaction are emergent. As with the child initiative, the influence of parent behaviors on the quality of adult generalized attachment representations (according to the AAI and ASA) was common (*k* = 5; Haydon et al., 2012; Massie & Szajnberg, 2002; Nivison et al., 2021; Roisman et al., 2002; Schoenmaker et al., 2015). While findings across studies were mixed, one study in particular (Schoenmaker et al., 2015) reports findings for both child and parent relational initiative predictors, with significance demonstrated only for the latter.

## Discussion

This scoping review provides a synthesis of longitudinal cohort studies that have explored correspondence between early relational health in primary care relationships (from the child’s birth to age three years) and subsequent outcomes of relationship quality across the life course. Most studies focused on prediction from infancy to early and middle childhood (56% and 28%, respectively). From here, study volume decreased incrementally into adolescence and adulthood with the latest follow-up at age 35 years. Of the studies examined, most focused on outcomes in parent–child, peer, and romantic relationships. Predictor measures of relational health in infancy primarily focused on either child or parent behaviors within observed dyadic interactions. Outcomes within eight relationship structures emerged: parent–child, sibling, familial, peer, child–teacher, romantic, generalized attachment, and general. Findings show that infant–parent relational health in the first three years of life plays a significant role in shaping both intra-familial and extra-familial relationships across development to young adulthood.

This review highlights consistent evidence for both direct and interactional influences of earliest relational health on later relationship outcomes across primary microsystems of development (family, friendships, and young adult relationships). These findings provide further support for the continuity of child–parent attachment during early and middle childhood developmental periods, and to broader forms of attachment in the adolescent and adult age groups. The findings also uniquely delineate the behavioral contribution that the child and parent make in shaping subsequent relational outcomes. Interestingly, decades long prospective findings from the Minnesota longitudinal study show infant attachment insecurity is associated with distinct emotion regulation strategies in adulthood 20–35 years later, with an identifiable pathway through friendship quality in childhood and adolescence (Girme et al., 2021). In general though, mature prospective cohort studies through to adulthood were rare, with only 18 unique studies identified. In other respects, some early studies (e.g., Grossmann et al., 2002) show a waning direct influence of early attachment by mid-adolescence. The expectation of a decreased direct signal from infant experience is highly reasonable, indeed expectable as life’s stressors and complexities accrue and impact multiple affiliative systems.

Discontinuity in attachment status over time is linked via specific risk exposures throughout development (e.g., child maltreatment, maternal depression, and conflicted family functioning; Becker-Stoll et al., 2008; Weinfield et al., 2000, 2004). Beyond the parent–child relationship, evidence is well established for the impact of exposure to negative parenting behavior during infancy and later problematic expectations of peer relationships, and functioning within them, across domains of victimization (Barker et al., 2008 [middle childhood]), bullying (de Vries et al., 2018 [middle childhood]; Ostrov et al., 2022 [adolescence]), and classroom aggression (Ostrov et al., 2022 [middle childhood]; Vitaro et al., 2006 [early childhood]).

The associated call to action for public health research is to pursue knowledge of developmental contexts in which secure trajectories may be diverted from their path, and equally mechanisms through which disorganized beginnings may become organized. Persistence with well-designed longitudinal studies is clearly needed, together with re-analysis of available data through a moderation lens.

## Limitations

Most notably, this review did not include studies of populations with clinical or developmental diagnoses; however, some of the comparison samples of the included studies provide support for the role of early parent–infant relational health in determining the relationship quality over time regardless of medical and developmental vulnerabilities (e.g., Doiron et al., 2022; Galán et al., 2017). It is also worth noting that, despite the stepwise grouping of both predictors and outcomes within each developmental period, there remained significant heterogeneity in the predictors examined as well as the measures used within outcome subgroups (validated versus study derived). Additionally, there was considerable variability in sample size across studies. This may in part contribute to variability in the significance observed across studies; equally, it may attest to the robustness of the construct of early relational health—no matter how operationalized and within which sampling frame, the direction of findings held. The potential influence of publication bias should also be considered in the context of findings presented here given null findings are typically underrepresented in published literature (Goldacre & DeVito, 2018).

## Research Gaps and Future Directions

Findings of this scoping review highlight several important gaps in the current evidence. Each signal a call for future investment in what appear to be highly relevant predictors for relationship quality across the life course. Some gaps reflect historic decisions, for example, to focus on outcome prediction from the infant–mother relationship; the impact of the fathers’ early attachment with the child on relational trajectories remains under-researched. More recent studies are beginning to show longitudinal influences of infant–father relational health across outcomes (e.g., Kochanska et al., 2015 [middle childhood]). While contemporary population cohort studies confirm strikingly similar infant attachment classification proportions by mother–infant or father–infant dyads (McIntosh et al., 2024), deliberate investment in infant–father prospective studies is needed. Moreover, only a few studies examined the place of kin and cultural connection for parent and child in direct and moderated pathways (as in Howes & Obregon, 2009). Investment in indicating socio-cultural context within future longitudinal cohort designs is key to understanding overt and subliminal protective influences on relational life.

Research examining links from infant relational beginnings to later relationships with siblings, family, and teachers is scarce across all developmental epochs. Those captured here all show significance across early and middle childhood (Carlson et al., 2004; Mintz et al., 2011; O’Connor et al., 2011).This lead seems important to pursue. Within the extra-familial ecology, exploration of the teacher’s relational initiative with children is equally emerging as critical, including the potential for the teacher–child relationship to compensate for early trauma in the parent–infant relationship, especially in the face of multiple adverse experiences and disadvantage (Jordan et al., 2014). Finally, no study has yet examined intergenerational continuities from children’s early relational experiences with their parents (from conception to age three) to the quality of their relational interactions with next-generation offspring. This provides an important direction for future research.

## Actionable Insights

In better understanding the influence of early relational health in the parent–child dyad on relationship quality within the broadening social ecology across the life course, we make the following recommendations on research investment and policy foci.

1. The existing literature highlights the centrality of early relational health between infants and carers in policy and practice settings. In particular, focus on early detection of and response to challenged relationship security appears paramount, especially in contexts of vulnerability to adverse life course outcomes.
2. It follows that laying strong foundations for parent–child relational health from birth requires knowledge and skill sets focused on early identification of and responses to maladaptive patterns of interaction to support recovery.
3. Beyond investment in individualized treatment, investment in community messaging about early relational health may raise awareness and mitigate risk across varying social ecologies.
4. There is a notable gap in studies that track developmental trajectories across diverse cultural backgrounds, care relationships, and kinship systems. In Australia, this especially includes relational pathways in collectivist First Nations communities.
5. Leveraging existing research investment in long-term cohort studies is essential, with expansion of their breadth and reach into intra- and extra-familial systems across the life course.

## Conclusion

Findings from this scoping review suggest that experiences in close relationships in the earliest years of the life course create a legacy that continues to shape relational functioning at least through to young adult life. It is possible, as cohort studies mature, that additional evidence across multiple generations might be reported. Indeed, some of the oldest studies of relational development may be able to answer such questions with available data now (i.e., Sroufe et al., 2009). However, based on the current literature reviewed alone, there is significant evidence to suggest that social policies that direct public health investments to promoting early relational health should yield dividends in relational health well beyond early childhood. These dividends may extend further as new data from old cohorts continues to be collected across the lifespan.

Public health investments should thus focus on protecting, nurturing, and strengthening the infant–parent attachment relationship, which is the most proximal of processes in human development, and which if supported to be secure, greatly enhances the child’s capacity to function well and to adapt to stress within their broadening social ecology, across their life course. Goffin et al. (2018) refer to a secure base from which to cooperate with others, and the early origins of a ‘willing stance’ to relationships. This summarizes in our view the essence of this brief review: that ongoing research investments are needed in areas most likely to support longevity in the growing child’s capacity to find restoration in close relationships and to hold willingness to contribute positively to interpersonal relationships, especially in the face of challenge.

Collectively, the findings of this review emphasize the importance of continued investment in public health approaches to promoting early relational health and the importance of rigorous research on the developmental determinants of secure attachment relationships. This knowledge will inform collective approaches to strengthening human relational trust at the very beginning of life. Research focused on maladaptive patterns evident in early parent–child interactions and interaction with accruing risk across time is key to protecting relational health in vulnerable contexts. Equipping universal health providers with knowledge of relational health identification and responses to trauma provides one foundation for supporting parent–child relational health from birth (Clancy et al., 2020; Opie et al., 2023). Such knowledge provides impetus for public health policy targeting positive development across the life course, in which investment in early relational health is recognized as key to the promotion of children’s positive relationship expectations and adaptation to increasingly complex social ecologies, across their lifetimes.

## Supplementary Information

Below is the link to the electronic supplementary material.

Supplementary file1 (DOCX 80 KB)

Supplementary file2 (DOCX 26 KB)

Supplementary file3 (DOCX 151 KB)
