# Supplementary material for: Mapping the Influence of Infant–Parent Relational Quality on Life Course Relationships: A Scoping Review of Prospective Cohort Studies
Source: Clin Child Fam Psychol Rev. 2025 Jun 8;29(2):213–28. doi: 10.1007/s10567-025-00527-5 (PMC13282218; doi:10.1007/s10567-025-00527-5)
Supplement: Supplementary file 2 — Supplementary file2 (DOCX 26 KB) [file 10567_2025_527_MOESM2_ESM.docx]

**Online Resource 2**

**Search Syntax for Database Search**

| Concept 1 – Outcome  Who/**What**: Child & Family Relational Ecology  Reflects child exposure to relationships and observations of relationships with and between parents, siblings, grandparents, and caregivers | Concept 2 – Outcome  When: First 3 years of life  Reflects the period from conception up to three years post-partum. | Concept 3 –  How |
| --- | --- | --- |
| **[ti,ab]**  (Infan* OR child* OR fetal OR fetus OR foetal OR foetus OR “parent-child” OR “child-parent” OR “parent-infant” or “infant-parent” OR “fetus-parent” OR “parent-fetus” OR “foetus-parent” OR “parent-foetus” OR “mother-child” OR “child-mother” OR “mother-infant” or “infant-mother” OR “fetus- mother” OR “mother-fetus” OR “foetus-mother” OR “parent-child” OR “father-child” OR “child-father” OR “father-infant” or “infant-father” OR “fetus-father” OR “father-fetus” OR “foetus-father” OR “father-foetus” OR  “maternal-child” OR “child-maternal” OR “maternal-infant” or “infant-maternal” OR “fetus-maternal” OR “maternal-fetus” OR “foetus-maternal” OR “maternal-foetus” OR “paternal-child” OR “child-paternal” OR “paternal-infant” or “infant-paternal” OR “fetus-paternal” OR “paternal-fetus” OR “foetus-paternal” OR “paternal-foetus” OR ”parental-child” OR “child-parental” OR “parental-infant” or “infant-parental” OR “fetus-parental” OR “parental-fetus” OR “foetus-parental” OR “parental-foetus”)  **N1** **(attach* OR relations* OR interact* OR bond*)**  OR  (Grandparent* OR grandmother* OR grandfather* OR parent* OR maternal OR paternal OR father* OR mother* OR caregiv* OR alloparent* OR cousin* OR Aunt* OR Uncle* OR Nanny OR Nannies OR “early childcare” OR “early childhood educator” OR friend* OR peer* OR playmate* OR ‘play mate*’ OR ‘class mate* OR classmate*) **N1** (**sensitivity OR involve* OR relation* OR interact* OR investment* OR bond* OR attach* OR availab* OR responsiv* OR warm* OR hostil* OR connect*)**  OR  (Brother OR Sister OR  Sibling) **N1 (Relations* OR conflict* OR interact*)**  OR  **“**dyadic synchrony”  OR “dyadic attunement”  OR “dyadic mutuality”  OR Kinship  OR    ‘Parent-parent’  OR Interparent*  OR “parenting”  OR “parent* styles”  OR “parent* quality”  OR “parent* behav*”  OR “parent* conflict”  OR coparent*  OR “co-parent*”  OR    “Family conflict” OR “Family cohesion” OR “Family relations*” OR “Family function*”  OR “Family interact*” OR “Family involve*”   OR    “Dyadic adjustment” OR “Relationship quality” OR “relationship conflict” OR “relationship satisfaction” OR “Marital quality” OR “marital conflict” OR “Marital satisfaction”  OR  “Social network analys*” OR  Egocentr* OR  “Whole network*” OR  “Network analys*” OR  “Ego network*” OR  Egonetwork* OR  Sociometr* OR  Sociogram* OR  Sociomap* OR  Sociocentr* OR  “Graph theory” OR  “Structural network*” | **[ti,ab]**  Perinatal* OR  Peri-natal  OR Neonatal*  OR Peripartum  OR Puerperium  OR Puerperal*  OR Postnatal*  OR Post-natal  OR Postpart*  OR Childbirth  OR Primipar*  OR “New parent*”  OR “Transition to parent*”  OR “Transition to mother*”  OR “Transition to father*”  OR Baby  OR Infan*  OR Birth  OR Antenatal*  OR Ante-natal  OR Antepartum  OR Pregnan*  OR “Peri-conception”  OR “Periconception”  OR Toddler*  OR “early years”  OR “first year of life”  OR “first 1000 days”  OR (“12 month*” or “12-month*” or “18-month*" or “18 month*” or “two years” or “two-years” OR "three years” OR “three-years” OR “2 years” OR “2-years” OR “3 years” OR “3-years”) N2 (age* OR “life” OR old) | **[ti,ab]**  Cohort OR  Longitudinal* OR  Prospective* OR  ‘Follow up’ OR  ‘Follow-up’ |
