# Supplementary material for: Mapping the Influence of Infant–Parent Relational Quality on Life Course Relationships: A Scoping Review of Prospective Cohort Studies
Source: Clin Child Fam Psychol Rev. 2025 Jun 8;29(2):213–28. doi: 10.1007/s10567-025-00527-5 (PMC13282218; doi:10.1007/s10567-025-00527-5)
Supplement: Supplementary file 3 — Supplementary file3 (DOCX 151 KB) [file 10567_2025_527_MOESM3_ESM.docx]

**Online Resource 3
Data Extraction Table and Reference List for all Included Studies**

| Author (Year), Country Study cohort | Analytic N (% male) | Predictor Age | Predictor Relationship/ Variable(s) | Predictor Measure(s) | Outcome Age | Outcomes  Relationship/ Variable(s) | Outcome Measure(s) | Key Findings |
| --- | --- | --- | --- | --- | --- | --- | --- | --- |
| Aviezer et al. (2002), Israel  The Haifa Longitudinal Study (Sagi et al., 1985) | 66 (50%) | 13-15 m. | *Parent-child*  1) Attachment | 1) SSP; obs. | MC: 11 y. | *General*  1) Contemporaneous representation of relationships  *Peer* 2) Social competence | 1) SAT; interview projective 2) Study derived; teacher report | *Child Initiative:* Correlation between infant-mother attachment  and SAT score approached sig (p<.01). Non sig correlation between infant-mother/infant-father attachment and social competence. |
| Barglow et al. (1998), USA  Follow-up study of original cohort (Barglow et al. 1987) | 84 (52%) | 12 m. | *Mother-infant*  1) Attachment (2-way) 2) Maternal sensitive responsiveness | 1) SSP; obs. 2) Self-descriptive Q-sort; maternal report | EC: 6-7 y. | *Peer*  1) Positive social interaction 2) Quality of social interaction | 1) Unstructured play; obs. 2) PIPS Test; obs. | *Child Initiative:* Attachment significantly predicted free play social competence in girls, such that secure attachment predicted better social competence. Attachment did not predict social competence in boys. *Parent Initiative:* Maternal sensitive responsiveness did not significantly predict social competence for boys or girls. |
| Bar-Haim et al. (2000), USA  Longitudinal project (Stifter & Fox, 1990) | 48 (48%) | 14, 24 m. | *Mother-infant*  1) Attachment | 1) SSP; obs. | EC: 58 m. | *Parent-child*  1) Attachment (ABC classifications) 2) Attachment representations; Emotional Openness | 1) Modified SSP; obs. 2) SAT; obs. | *Child Initiative:* No stability of attachment (ABC class.) between 14 and 58 m., or between 24 and 58 m. No sig. group effects for 14 or 24 m. attachment (ABC class.) with 58 m. MR or EO.  Children in the ‘stable-secure’ group (i.e., security between infancy and 58 m.) showed greater EO compared to children with unstable group. There was no significant difference between the groups in the index of MR. |
| Barker et al. (2008), Canada  Quebec Longitudinal Study of Child Development | 1970 (51% boys) | 17 m. | *Mother-infant*  1) Harsh reactive parenting | 1) PACOTIS (harsh/reactive subscale); parent-report | MC (trajectory): Kindergarten-first grade | *Peer*  1) Peer victimization | 1) Study-derived; maternal-report (kindergarten), teacher-report (1st grade) | *Parent Initiative:* Sig association between harsh reactive parenting and victimization. Three risk variables differentiated those in the high/chronic victimization group (i.e., high victimization at kindergarten and 1st grade) from those in the low/increasing group: insufficient family income; harsh, reactive parenting; and greater physical aggression in the child. |
| Barry et al. (2010), USA  Family Study | 102 families (50% boys) | 7, 15 m. | *Parent-child*  1) Child-parent Affection 2) Child-parent Joy 3) Child-parent Anger | 1/2/3) Parent-child interaction; obs. | EC: 52, 67 m. | *Parent-child*  1) Child-parent Affection 2) Child-parent Joy 3) Child-parent Anger | 1/2/3) Child’s and parents’ positive and negative emotions during interactions; obs. | *Child Initiative:* Children’s anger was highest at 15 m. and decreased linearly over time thereafter. Children's joy, particularly with mothers, increased over time. No pattern of change in children's affection with parents. |
| Becker-Stoll et al. (2008), Germany  Regensburg Longitudinal Study | 43 (37%) | 12, 18 m. | *Mother-infant*  1) Attachment | 1) SSP; obs. | A: 16 y. | *Parent-child*  1) Attachment representation 2) Dyadic interaction | 1) AAI & AQS; interview, obs. 2) Task-oriented dyadic interaction; obs. | *Child Initiative:* No sig continuity in attachment from 12 m. to 16 y. -- discontinuity was linked to a higher amount of experienced risk factors Sig continuity in infant-mother attachment behavior and interaction behavior towards mother at age 16. Sig association between infant attachment and adolescent autonomy and relatedness behavior. |
| Bedford et al. (2017), USA  Durham Child Health and Development Study (DCHDS) | 206 | 6 m. | *Mother-infant*  1) Infant/parent gaze 2) Maternal sensitivity | 1) FFSFP; obs. 2) Free play; obs. | MC: 7 y. | *General*  1) Callous unemotional behaviors | 1) ICU; maternal-report | *Child x Parent Initiative*: Interaction between infants’ mother- directed gaze and maternal sensitivity sig predicted CU behaviors -- neg association between infant gaze and later CU behaviors only for those with low maternal sensitivity. No significant indirect effects were found for either infants’ mother-directed gaze, or maternal sensitivity, to CU behaviors via emotion recognition. |
| Beijersbergen et al. (2012), Netherlands  Longitudinal study (Jaffari-Bimmel et al., 2006) | 125 | 12 m. | *Mother-infant*  1) Attachment 2) Maternal sensitivity | 1) SSP; obs. 2) Free play; obs. | A: 14 y. | *General*  1) Attachment | 1) AAI; interview | *Parent Initiative:* High levels of maternal sensitive support 12 m. predicted continuity of secure attachment. *Child x Parent Initiative:* A relative increase in maternal sensitive support from 12 m. to adolescence predicted children’s change from insecurity in infancy to security at 14 y. No continuity of secure or insecure attachment from 1 to 14 y. without interaction of maternal sensitivity. |
| Berlin et al. (1995), USA  Pennsylvania Child and Family Development Project | 64 (56%) | 12 m. | *Mother-infant*  1) Attachment | 1) SSP; obs. | EC: 5-7 y. | *Peer*  1) Loneliness | 1) LSDS; self-report | *Child Initiative:* Children classified insecure-ambivalent at 12 m. reported most loneliness. Children classified insecure-avoidant in infancy reported least loneliness. Children classified secure reported intermediate loneliness. |
| Blandon et al. (2010), USA  Longitudinal cohort (Calkins, Dedmon, Gill, Lomax, & Johnson, 2002) | 253 (46%) | 24 m. | *Mother-infant*  1) Maternal control 2) Maternal positive behavior | 1/2) Teaching, free-play, compliance tasks; obs | EC: 5 y. | *Peer*  1) Perceived peer acceptance 2) Social skills 3) Problem behavior 4) Likeability & Aggressions | 1) PSPCSA; projective 2/3) SSRS; teacher-report 4) Sociometric ratings; peer report | *Parent Initiative:* Maternal control positively associated with perceived peer acceptance, problem behavior, and children’s aggression; negatively correlated with maternal positive behavior, social skills, and children’s likeability. Maternal positive behavior positively associated with social skills; negatively associated with negativity and perceived peer acceptance. |
| Blume et al. (2022), USA  Fragile Families and Child Wellbeing Study (FFCWS) | 4374 (52% boys) | 36 m. | *Parent-child*  1) Supportive parenting | 1) HOME; obs. | EC: 5 y. MC: 9 y. | *Peer*  1) Pro-social behaviors | 1) ASBI (interview); parent-report | *Parent Initiative:* Sig. positive correlation between supportive parenting and prosocial behaviors at 5 and 9 y. No support for parent-driven model (SEM) - supportive parenting behaviors did not sig alter patterns of later child pro-social behavior. |
| Boldt et al. (2016), USA  Family Study | 15 m.: 101 families (49%) 100 m.: 87 families (53%) 123 m.: 82 families (55%) | 1) 15 m.  2) 25 m. 3) 25 m. 4) 7, 15, 25 m. | *Parent-child*  1) Attachment security 2) Parent-rated security 3) Observer rater security 4) Mother/father responsiveness | 1) SSP; obs 2/3) AQS; obs 4) Naturalistic parent-child interactions; obs. | 1) MC: 8, 10 y. 2) MC: 10 y. | *Parent-child* 1) Child reported security 2) Attachment behavior | 1) KSS (8 y.), KKS & PIML (10 y.); interview, self-report 2) IABC; obs. | *Child Initiative:* For mother-child dyads, IABC Security was positively associated with observer-reported AQS security scores at 25 m. For father-child dyads, IABC Security was positively associated with observed-rated AQS security at 25 m. IABC Avoidance was negatively related with both father- and observer-rated AQS at 25 m.  *Parent Initiative:* SEM - history of responsive care was meaningfully associated with Security, Avoidance, and Disorganization at age 10, in both mother-child and father-child relationships; most recent care uniquely predicted Security. |
| Boldt et al. (2020), USA  Family Study | 25 m.: 100  38 m.: 100  10 y.: 82  12 y.: 79 families | 25 m. 38 m. | *Parent-child*  1) Attachment security | 1) AQS; obs. | MC: 10, 12 y. | *Parent-child*  1) Regulation of negative emotional tone  *General* 2) Regulation of negativity | 1) Study-derived; obs. 2) CSI-4, ASI-4 (ODD subscales); parent-report | *Child Initiative:* Pairwise correlations - attachment security at 2 y. was positively associated with children's regulation of negative emotional tone in interactions with parents, and regulation of negativity in social interactions at 10-12 y. of age. |
| Borghini et al. (2018), Switzerland  Longitudinal study on CLP children | 46 (32 Cleft lip palette, 14 control) | 12 m. | *Mother-infant*  1) Attachment | 1) SSP; obs. | EC: 4 y. | *Parent-child*  1) Attachment | 1) ASCT; projective | *Child Initiative:* Attachment categories for the whole sample were not stable across time (only 35% of the categories at 12 m. old were the same at 4 y. - with 12 dyads secure-secure from 12 m. to 4 y.). |
| Bornstein & Putnick (2021), USA  13-year longitudinal study | 255 mother- child dyads | 5 m. | *Mother-infant*  1) Relationship quality | 1)EAS (3rd ed.); obs. | 1) EC: 4 y. | *Mother-child*  1) Relationship quality | 1) Mother, Child, and Dyadic TTS; obs. | *Parent Initiative:* Mother– child dyadic relationship quality was moderately to strongly stable from 5 m. to 4 y. Longitudinal stabilities did not differ by child genders. |
| Bosquet et al. (2006), USA  Minnesota Longitudinal Study of Parents and Children (not reported) | 267 high-risk | 12, 18 m. | *Mother-infant*  1) Attachment | 1) SSP; obs. | 1) MC: 6th grade | *Peer*  1) Relationship representations | 1) Battery of narrative projective story telling tasks (2 from TED; 2 from the TAT), a sentence completion task, friendship interview | *Child Initiative:* Insecure attachment relationships in infancy predicted negative peer relationship representations in 6th grade. No sig associations between insecure attachment history and relationship representation (total). |
| Boutwell et al. (2012), USA  Early Childhood Longitudinal Study, Birth Cohort (ECLS-B) | NR - sample of twin pairs drawn from the ECLS-B | 24 m. | *Mother-infant*  1) Maternal disengagement (derived from attachment) | 1) TAS-45); obs. | EC: 4 y. | *General*  1) Externalizing behavioral problems (aggressive, impulsive, destructive actions) | 1) PKBS-2; parent-report | *Parent Initiative:* Sig genetic x environment interaction - maternal disengagement moderates the influence of genes on the emergence of childhood externalizing problems (genetic influences on antisocial behavior become more pronounced as levels of maternal disengagement increase). |
| Burgess et al. (2003), USA  Not reported | 172 | 14 m. | *Mother-infant*  1) Attachment | 1) SSP; obs. | EC: 4 y. | *Peer*  1) Social reticence 2) Shyness 3) Sociability 4) Externalizing behaviors | 1) Child-peer interactions, obs.  2/3) CCTI; parent-report 4) CBCL (aggressive behavior and conduct problems subscales); parent-report | *Child Initiative:* Infants who had an avoidant attachment with their mothers had more aggressive behaviors at age 4 than either securely or ambivalently attached infants. This predictive relation was qualified by interaction of avoidant attachment and uninhibited temperament. Non-sig relationship between attachment and social reticence. |
| Cao et al. (2021), China  Not reported | 92 (48%) | 24 m. | *Mother-infant*  1) Child's connectedness behavior 2) Maternal encouragement of connectedness | 1/2) Naturalistic parent-child interactions; obs. | MC: 7 y. | *Peer*  1) Peer liking | 1) Child-peer interaction (lab); obs & interview | *Child Initiative:* Sig negative association between children’s connectedness behaviors at 2 and peer liking at 7 y.  *Parent Initiative:* Sig positive association between maternal encouragement of connectedness when children were 2 and peer liking at 7 y.. |
| Carlson (1998), USA  Minnesota Mother–Child Project (Egeland & Brunnquell, 1979) | 157 (59%) | 12, 18 m. | *Mother-infant*  1) Attachment | 1) SSP; obs. | A: 13 y. | *Mother-child*  1) Parent-child relationship quality | 1) Parent-child interaction (lab); obs. | *Child Initiative:* No sig. correlation between, infant history of attachment disorganization and parent-child boundary dissolution at 13 y.. |
| Carlson et al. (2004), USA  Minnesota Mother–Child Project; Egeland & Brunquell, 1979) | 185 (55%) | 1) 12, 18 m. 2) 24 m. | *Mother-infant*  1) Attachment  2) Relationship quality | 1) SSP 2) Problem solving procedure; obs | 1) EC: 4.5 y. 2) MC, A: 8, 12 y. 3) EC, MC, A: 5, 8, 12 y. | *Peer*  1) Peer relationships  *Parent-child* 2) Relationship representational organization  *Peer* 3) Peer competence rankings | 1) PIPS Test; obs. 2) Family drawings, Battery of narrative projective tasks; projective 5) Study derived; teacher report | *Child Initiative:* Sig positive correlations between infant attachment and peer relationships at 4.5 y., family relationship representation at 8 y., peer competence at 8 and 12 y., peer relationships at 12 y. No sig correlation between infant attachment and peer competence at 5 y. Sig correlations between 24 m. attachment and all relevant outcome variables. |
| [Cicchetti and Barnett (1991), USA  Harvard Child Maltreatment Project](https://www.tandfonline.com/doi/full/10.1080/14616734.2020.1800769) | 66 children (36 (55%) maltreated; 30 (53%) comparison) | 36 m. | *Mother-infant*  1) Attachment | 1) SSP; obs. | EC: 4 y. | *Parent-child*  1) Attachment | 1) Modified SSP; obs. | *Child initiative:* Maltreated - From 36- and 48-m., 33% of securely attached children remained stable, 50% of insecurely attached children retained insecurity. Non-maltreated - From 36- and 48-m., 93% of securely attached remained stable, 16% of insecurely attached children retained insecurity. Sig not reported. |
| Célia et al. (2018), Canada  The Concordia Longitudinal Risk Project | 56 mother-infant dyads (41%) | 6, 12, 18 m. old | *Mother-infant*  1) Child Emotional availability (responsiveness and involvement) | 1) EAS (2nd ed.); obs. | EC: 55 m. | *Parent-child*  1) Child EA (child responsiveness and involvement) | 1) EAS (2nd ed.); obs. | *Child Initiative:* Sig within-person and between-person variation in child responsiveness and involvement (average interindividual stability according to ICC coefficients was small for both). Sig increase in child EA from infancy to 55 m. No variability around rate of change, but sig variability around the initial status for responsiveness and involvement. |
| Chen et al. (2002), China  Not reported | 172 children (46%) | 24 m. | *Mother-infant*  1) child non-compliance | 1) Compliance tasks; obs | EC: 4 y. | *Peer*  1) verbal aggression 2) physical aggression | 1/2) Child-peer interaction (lab) obs. | *Child Initiative:* Sig positive correlation between 24 m. child noncompliance and both verbal and physical aggression at 4 y. |
| Cyr et al. (2014), USA  the Early Parenting Project | 82 mother–child dyads (45 % boys) | 12 m. | *Mother-infant*  1) Attachment | 1) SSP; obs. | MC: Summer following 1st grade | *General*  1) Child aggression | 1) CBCL (aggressive behavior subscale); maternal report | *Child Initiative:* Sig negative correlation between infant attachment and child aggression in 1st grade. |
| Dallaire et al. (2005), USA  NICHD Study of Early Childcare (NICHD SECCYD) | 99 mother–child dyads | 6, 15, 24 m. | *Mother-infant*  1) Attachment 2) Maternal sensitivity | 1) SSP; obs. 2) Study-derived; obs | EC: 6 y. | *Parent-child*  1) Separation anxiety | 1) CPI; structured interview | *Child Initiative:* Attachment security at 15 m. was associated with lower levels of child separation anxiety.  *Parent Initiative:* Maternal sensitivity at 6 m., 15 m., and 24 m. associated with lower levels of child separation anxiety. |
| Dantchev et al. (2019), UK  Avon Longitudinal Study of Parents and Children (ALSPAC) | 6838 | 8, 33 m. | *Mother-infant*  1) Maternal bonding | 1) Study-derived; parent-report | A: 12 y. | *Sibling*  1) Sibling bullying | 1) OBVQ; child-report | *Parent Initiative:* Children with higher levels of maternal bonding at infancy were less likely to be bullied by their siblings and/or be a victim of bullying by their siblings (relative to children who were not involved in bulling as either a perpetrator or victim) |
| de Vries et al. (2018), Holland  Generation R Study (Jaddoe et al. 2012) | 1298 | 36 m. | *Parent-child*  1) Parental hostility 2) Harsh disciplinary practices | 1/2) Dutch version of the BSI (hostility subscale); parent-report | MC: 7.5 y. | *Peer*  1) Bullying | 1) PEERS Measure; child-report | *Parent Initiative:* Parental hostility and harsh disciplinary parenting practices at 3 y. were positively associated with children's bullying at 7 y. |
| Degnan et al. (2015), USA  Not reported | 315 (48%) | 1/2) 9 3) 36 m. | *Parent-child*  1) Infant avoidance 2) Maternal sensitivity 3) Maternal gentle discipline | 1) Lab-TAB (Goldsmith & Rothbart, 1999); obs. 2) Parent-child interaction (home); obs.  3) 5 minutes clean up episode (home); obs. | EC: 48, 60 m. | *Peer*  1) Social reticence | 1) Peer free-play; obs. | Three longitudinal risk trajectories of social reticence behavior were determined: a High-Stable trajectory, a High-Decreasing trajectory, and a Low-Increasing trajectory.  *Parent x Child Initiative:* Infants displaying greater avoidance, 9-month maternal sensitivity and 36-month maternal gentle discipline were both positively associated with membership in High-Stable, compared to High-Decreasing. Infants displaying lower avoidance, maternal sensitivity was positively associated with membership in High-Decreasing, compared to Low-Increasing.  *Parent Initiative*: Maternal sensitivity was positively associated with High-Stable trajectory when Maternal Gentle Discipline was lower. |
| Doiron et al. (2022), Canada  Concordia Longitudinal Research Project | 163 infant-mother dyads | 6, 12, 18 m. old | *Mother-infant*  1) Co-regulation | 1) Free play (home); obs. | EC: 48 m. | *Parent-child*  1) Co-regulation | 1) Free play (home); obs. | *Parent Initiative: S*ymmetrical co-regulation in full-term and VLBW/preterm dyads followed a statistically significant cubic (s-shaped) trend from 6- to 48 m. Co-regulation trajectory followed a sig positive linear trend among the psychosocially at-risk dyads from 6- to 48-m.. |
| Easterbrooks et al. (2000), USA  N/A | 45 children (58%) | 18 m. | *Mother-infant*  1) Attachment | 1) SSP; obs. | MC: 7 y. | *Parent-child*  1) EA | 1) Dyadic interactions (lab, coded using EAS); obs | *Child Initiative:* Infant attachment security was related to child responsiveness and involvement at age 7. Greatest differentiation was found between infants with secure attachments and those with insecure- disorganized attachments. |
| Englund et al. (2011), USA  Minnesota Longitudinal Study of Risk and Adaptation (MLSRA; Sroufe et al., 2005) | 157 (50%) | 12, 18 m. | *Mother-infant*  1) Attachment | 1) SSP; obs. | 1) MC: 7,8,9 y. (composite) 2) A: 13 y. 3) A: 16 y. 4) AH: 23 y. | *Peer*  1) Peer competence  *Parent-child* 2) Parent-child relationship  *Peer* 3) Friendship security  Romantic 4) Relationship effectiveness | 1) Study-derived; teacher-report  2) Parent-child interactions; obs.  3) Current, non-romantic closest friend relationships; semi-structured interview 4) Relationship effectiveness scale; self-report | *Child Initiative:* Sig correlation between infant attachment and friendship security at 16 y. Sig. correlation between infant attachment and relationship effectiveness at 23. No sig correlation between infant attachment and peer competence in childhood. No sig correlation between attachment and parent-child relationships at 13 y. Path analysis - direct effect of infant attachment security and relationship effectiveness at 23 y. approached sig (p=.10). |
| Ensor et al. (2010), UK  Not reported | 88 mothers | 30 m. | *Mother-infant*  1) Positive and negative parenting | 1) PCIS (Deater-Deckard, Pylas, & Petrill, 1997); obs. | EC: 4 y. | *Peer*  Prosocial behaviors 1) willingness to share; 2) sharing/helping | 1) Willingness to share (adapting procedures developed by Moore et al., 1998); obs. 2) Child-peer interaction (adapted procedures developed by Hay et al., 1999); obs. | *Parent Initiative:* Higher levels of negative parenting were significantly correlated with lower levels of willingness to share for young mother families, but not for older mother families. |
| Ettekal et al. (2020), USA  Longitudinal study (Eiden et al., 2014) | 216 mother–child dyads (49% boys) - low income | 24 m. | *Mother-infant*  1) Maternal sensitivity | 1) Parent–Child ERA; obs. | EC: 4 y. MC: 2nd grade | *General*  1/2) Conduct problems (aggressive behavior) | 1) CBCL (aggressive behaviors subscale); maternal report 2) BASC-3 (aggression subscale); mother- and teacher-report | *Parent Initiative:* Maternal sensitivity at 24 m. did not sig correlate with conduct problems (aggressive behavior) at any age. No individual paths were statistically sig. |
| Feldman et al. (2013), Israel  Not reported | 68 | 5, 34 m. | *Parent-child*  1) Dyadic Reciprocity | 1) Parent-child interactions (coded using CIB manual); obs. | A: 13 y. | *Peer*  1) Dialogical Skills | 1) Child-peer interactions (coded using CIB manual); obs. | *Child Initiative:* SEM - maternal and paternal reciprocity were each uniquely predictive of social competence and lower aggression in preschool, which, in turn, shaped dialogical skills at 13 y.. |
| Fihrer et al. (2009), Australia  Not reported | 75 | 15 m. | *Mother-infant*  1) Attachment | 1) SSP; obs. | EC: 6-8 y. | *Family*  1) Attachment | Family drawing; projective | *Child Initiative:* Infant-mother attachment not related to family drawing. |
| Fish (2004), USA  Longitudinal study (Fish, 1998, 2001) | 82 low SES mother-infants dyads | 15 m. | *Mother-infant*  1) Attachment | 1) SSP; obs. | EC: 4 y. | *Parent-child*  1) Attachment | 1) SSP | *Child Initiative:* Infant attachment security was likely to be maintained, and about half of infants classified insecure changed to a secure classification by 4 y., demonstrating low but significant stability in attachment at the level of secure-insecure. |
| Galán et al. (2017), USA  Pitt Mother and Child Project | 187 | 18 m. | *Mother-infant*  1) Punitive Discipline | 1) Free play, clean up session (lab; coded with the Early Parenting Coding System), obs. | 1) MC: 10 2) A: 17 3) AH: 20 4) AH: 22 y. | *Peer*  1) Maladaptive SIP  *General* 2) Attitudes toward violence 3/4) Aggressive behavior | 1) Social vignette procedure 2) ATVC; self-report 3/4) SRDS; self-report | *Parent Initiative:* Sig interaction of MAOA allele and punitive discipline - boys with low activity variant who experienced more punitive discipline at 1.5 generated more aggressive responses to perceived threat at 10 y. relative to men with the high activity variant. Indirect effect of punitiveness on adult arrest records via aggressive response generation at 10 (African American subsample only). Association from punitive discipline to age 17 violent attitudes approached significance (p<.10). Association between MAOA x punitiveness and antisocial behavior in early adulthood mediated by maladaptive SIP. |
| Girme et al. (2021), USA  MLSRA | 102 | 12, 18 m. | *Mother-infant*  1) Attachment | 1) SSP; obs. | 1) AH: 20, 23, 26, and/or 35 y. | *Romantic*  1) relationship conflict | 1) Partner interaction (lab); obs. & interview | *Child Initiative:* Compared to stable secure infants (secure at 12 and 18 m.), stable insecure infants (insecure at 12 and 18 m.) displayed worse balanced-regulation and greater hypo-regulation strategies, and unstable insecure infants (insecure at 12 or 18 m.) displayed greater hyper-regulation strategies, in relationship-threatening situations 20 -35 y. later. |
| Gloger-Tippelt et al. (2002), Germany  Longitudinal study at the University of Heidelberg | 28 low risk families | 13 m. | *Mother-infant*  1) Attachment | 1) SSP; obs. | EC: 6 y. | *Parent-child*  1) Attachment | SCPDP (McArthur Story-Stem Battery); obs. | *Child Initiative:* Sig continuity of attachment from 1 to 6 y. of age (according to twofold secure/insecure distinction) |
| Godleski et al. (2019), USA  Infant Child Development project | 227 high risk families (51%) | 24 m. | *Mother-infant*  1) Maternal harshness 2) Maternal warmth/sensitivity | 1/2) Parent-child interactions (coded using 5-point scales; Clark, Musick, Scott, and Klehr, 1980; Clark, 1999); obs. | A: 8th grade | *General*  1) Rejection sensitivity | 1) CRSQ; self-report | *Parent Initiative:* Maternal harshness was predictive of rejection sensitivity in adolescence. No sig correlation between maternal warmth/sensitivity and rejection sensitivity. |
| Goffin et al. (2018), USA  Family Study; Play Study | 6.5 y.: 90 12 y.: 79 | 24 m. | *Parent-child*  1) Attachment | 1) AQS; obs. | 1) EC: 6.5 y. 2/3) A: 12 y. | *Parent-child*  1) willing stance  *General* 2) antisocial behavior problems 3) positive mutuality regarding conflicts | 1) Parent-child interaction (naturalistic); obs.  2) ASI-4; maternal- & paternal-report 3) Coded interactions of "hot button" issues; obs. | *Child Initiative:* Security at age 2 positively associated with willing stance at age 6.5 and with positive mutuality regarding conflicts at age 12, and negatively associated with child antisocial behavior problems at age 12 for both mother- and father-child associations. |
| Goldberg et al. (2019), USA  Fragile Families and Child Wellbeing | 2340 | 36 m. | *Parent-child*  1) Harsh parenting (psychological aggression; physical aggression) | 1) CTSPC (psychological aggression and physical subscales); parent-report | A: 15 y. | *Romantic*  1) Relationship instability 2) Relationship general quality 3) Physical IPV perpetration and victimization | 1) Study derived; self-report 2) Overall quality using a 5-point scale (poor to excellent); self-report 3) CTS; self-report | *Parent Initiative:* Harsh parenting at age 3 was not sig associated with risk of no relationship formation by 15 y., relative to three or more relationships. Associations comparing one to two lifetime relationships with three or more relationships were also non sig. |
| Grossmann et al. (2002), Germany  Bielefeld Longitudinal Study | 49 (53%) | 1) < 12 m. 2) 12 / 18 m. 3/4) 24 m. | *Parent-child*  1) Fathers sensitive responsiveness 2) Attachment 3) Mothers sensitive and interactive play 4) Fathers sensitive and interactive play | 1) Parent-child interactions (home); obs. 2) SSP; obs.  3/4) Dyadic free play (coded using SCIP scale); obs. | 1) EC: 6 y. 2) MC: 10 y. 3) A: 16 y. | *Parent-child*  1) Attachment security 2) Attachment representations  *General attachment*  3) Attachment representations | 1) SAT; interview projective 2) CAI; interview 3) AAI; interview | *Child Initiative: Sig associations between SSP attachment* and SAT security at age 6. Infant–mother attachment predicted attachment representations at age 10. Attachment quality was not associated with attachment at 16 y.  *Parent Initiative:* Attachment representations at 16 y. were predicted by fathers’ play sensitivity only. Mothers’ ratings on the SCIP scale at 24 m. was not significantly associated with any measure of children’s security of attachment representation. Fathers’ SCIP scores sig correlated with child CAI security at 10, and AAI security at age 16. Fathers’ SCIP scores negatively correlated with child CAI dismissing at 10 y. No sig correlation of parenting quality and AAI preoccupation at age 16. |
| Hamilton (2000), USA  Family Lifestyles Project (FLS) | 30 | 12 m. | *Parent-child*  1) Attachment | 1) SSP; obs. | A: NR | *General attachment*  1) Attachment representations | 1) AAI; interview | *Child Initiative:* Overall stability of secure versus insecure classifications was 77%. Infant attachment was sig predictor of adolescent attachment. |
| Harris et al. (2021), Canada  Not reported | 91 | 18 and 36 m. | *Mother-infant*  1) Child EA | 1) EAS (child subscales; 4th ed.); obs. | EC: 60 m. | *Parent-child*  1) Child EA | 1) EAS (child subscales; 4th ed.); obs. | *Child Initiative:* Sig positive fixed slope between child EA from 18 to 60 m. postpartum. |
| Haydon et al. (2012), USA  MLSRA | 112 | 1) 12 m. 2) 24 m. | *Parent-child*  1) Attachment 2) Parenting quality | 1) SSP; obs. 2) Problem solving procedure (lab); obs | 1) A: 16 y. 2/3/4/5) AH: 20-21 y., 26-28 y. | *Peer*  1) Friendship quality  *Romantic* 2) Relationship conflict  *General attachment* 3) Relationship satisfaction 4) Romantic attachment 5) Generalized attachment representation | 1) Study-derived; interview 2/3/4) Couples assessment in laboratory; individual interviews including the CRI, and then joint interaction; the Markman-Cox procedure and Ideal couple Q-sort. 5) AAI; interview | *Child Initiative:* 12 m. attachment positively associated with relationship satisfaction only. *Parent Initiative:* Moderate positive correlation of 24 m. parenting quality with adolescent friendship quality, adult relationship secure base processing, conflict resolution skills, romantic and generalized attachment; moderate negative correlation with adult romantic functioning. |
| Hedenbro & Rydelius (2014), Sweden  N/A | 15 families | 1) 3 m., 9 m., 18 m. | *Parent-child*  1) Attachment | 1) LTP; obs. | EC: 4 y. | *Peer*  1) Peer and social competence | 1) PBQ; teacher report | *Child Initiative:* Child's turn-taking initiative in the child–mother–father triad at 9 m. associated with better peer competence and social competence at 4 y. |
| Heuser et al. (2018), Germany   Bavarian Longitudinal Study | 1147 | 5 m. | *Parent-child*  1) Parent-infant relationship | 1) Interview; parent-report 1) Parent-child interaction; nurse obs. | 1) EC: 6 y. 2) MC: 8 y. | *Peer*  1/2) Friendship | 1) FFI (semi-structured); child report 2) Mannheimer Parent Interview (contact with peers subsection); parent report | *Parent Initiative:* Better parent-infant relationships predicted having more friends at 8 y. of age and being more accepted at 6 y. of age (parent report). Better parent-infant relationships, higher cognitive abilities, and fewer motor and behavioral problems predicted more friendships and higher peer acceptance (after adjusting for covariates). |
| [Howes and Hamilton (1992), USA  Not reported](https://www.tandfonline.com/doi/full/10.1080/14616734.2020.1800769) | 72 (68%) | 12 m. | *Mother-infant*  1) Attachment | 1) SSP; obs. | EC: 48 m. | *Parent-child*  1) child-parent attachment  *Child-teacher* 2) child-teacher attachment | 1) SSP; obs. 2) AQS; obs. | *Child Initiative:* 72% of the children had the same attachment classification at 12 and 48 m. Secure (89%) and ambivalent (73%) classifications were more stable than avoidant classifications (24%). No overall concordance between 12-month maternal attachment and child's 4-year-old teacher attachment classification. |
| Howes et al. (2011), USA  Early Head Start Research and Evaluation Project | 88 (50%) | 14, 24 m. | *Parent-child*  1) Attachment | 1) AQS; obs. | EC: 54 m. | *Parent-child*  1) Attachment behavior and representations | 1) Story stem interview; obs. | *Child Initiative:* AQS security scores at 14 m. were positively associated with Story Stem narrative coherence scores. AQS security scores were not associated with Security, Deactivation and Emotional Integration on Story Stem. |
| Hubbs-Tait et al. (1994), USA Longitudinal study (Hann et al., 1994) | 44 adolescent mother-infant dyads | 13 m. | *Mother-infant*  1) Attachment | 1) SSP; obs. | EC: 54 m. | *General*  1) Social skills, behavior problems | 1)CBCL (externalizing social subscale); maternal report | *Child Initiative:* Infant-mother attachment 14% of the variance in externalizing behavior problems. Attachment did not show any significant predictive value for later social skills. |
| Jacobsen et al. (2000), Germany | 33 | 9–14, 15-19 m. | *Mother-infant*  1) Attachment | 1) SSP; obs. | EC: 72 m. | *Parent-child*  1) Attachment | 1) Modified SSP; obs. | *Child Initiative:* Sig relation between infant attachment disorganization and disorganization at 6 y.. |
| [Jacobsen et al. (1997), Germany Longitudinal study (Ziegenhain & Jacobsen, 1999)](https://www.tandfonline.com/doi/full/10.1080/14616734.2020.1800769) | 32 | 12, 18 m. | *Mother-infant*  1) Attachment | 1) SSP; obs. | EC: 6 y. | *Parent-child*  1) Attachment | 1) Modified SSP; obs. | *Child Initiative:* 63% maintained either a secure or an insecure attachment pattern, and 50% maintained the exact same attachment pattern from 12 m. to 6 y. 84% maintained either a secure or an insecure pattern; 66% maintained the exact same pattern from 18 m. to 6 y. Longitudinal relations between separate attachment groups were sig from both 12- and 18-m. to 6 y.. |
| Kim et al. (2014), USA  Family Study | 87 (53%) | 15 m. | *Parent-child*  1) Attachment | 1) SSP; obs. | MC: 80, 100 m. (composite) | *General*  1) Antisocial behavior | 1) ICU & CSI-4 (composite); parent report | *Child Initiative:* Security with either parent had no main effect on the child’s antisocial behavior. Moderated mediation - links between low tense discomfort and future antisocial behavior in insecure parent-child dyads were mediated by parental stronger discipline pressure. |
| Kim et al. (2017), USA  Family Study | 67 m.: 92 80 m.: 90 | 1) 15 m. 2) 25, 38, and 52 m. (composite) | *Parent-child*  1) Attachment 2) Empathy | 1) SSP; obs. 2) Scripted simulated distress paradigm | EC: 67, 80 m. | *Peer*  1) Prosociality | 1) HBQ (prosocial behavior scale); maternal- & paternal-report | *Child Initiative:* Security moderated the path from empathy to prosociality (in mother- and father-child dyads). For insecure children, but not secure ones, variations in empathy related to prosociality (insecure and unemphatic children were low in prosociality) |
| Kochanska et al. (2008), USA  Family Study | 52 m.: 99 67 m.: 91 | 1) 7, 15 m. 2) 25-38 m. | *Mother-infant*  1) Maternal responsiveness 2) Child responsive stance toward mothers | 1/2) Parent-child interactions (naturalistic); obs. | 1) EC: 52 m. 2) EC: 67 m. | *General*  1) Conscience 2) Disruptive behavior | 1) Sorting task vs playing with toys, observed rule-compatible behavior playing a game (lab), picture stories; obs. 2) CSI-4; maternal- & paternal-report | *Child Initiative:* Association between children's responsive stance toward their mothers at 3 y. and children’s manifestations of oppositional, defiant, callous, and antisocial traits at 6 was fully mediated by conscience at preschool age. *Parent Initiative:* Maternal responsiveness in infancy predicted child adoption of a responsive stance toward the mother at the toddler age, in turn linked to formation of conscience. |
| Kochanska et al. (2012), USA  Parent-child Study & Family Study | Parent-child study, 73 m.: 48 Family study, 80 m.: 90 (52%) | 14, 15 m. (Parent-child study, Family study) | *Parent-child*  1) Attachment | 1) SSP; obs. | EC: 73, 80 m. (Parent-child study, Family study) | *General/Peer* 1/2) Antisocial outcomes | 1) ICU, CSI-4, HBQ; maternal- & paternal-report 2) PBQ; teacher-report | *Child Initiative:* Moderation - in insecure dyads, a pattern of coercion emerged between children who were anger prone as toddlers and their parents, resulting in parents’ increased power-assertive discipline. Power assertion in turn predicted children’s rule-breaking conduct and a compromised capacity to delay in laboratory paradigms, as well as oppositional, disruptive, callous, and aggressive behavior (teacher- and parent-report). This causal chain was absent in secure dyads. |
| Kochanska et al. (2015), USA  Family Study | 52 m.: 99 67 m.: 92 10 yrs.: 82 | 25 m. | *Parent-child*  1) Attachment security | 1) AQS; obs. | 1 /2) MC: 10 y. | *Parent-child* 1) Obligation to obey mothers/fathers 2) Perceived trustworthiness of mothers and fathers as attachment figures | 1) Strategic Disclosure Questionnaire; interview  2) PIML; interview | *Child Initiative:* Attachment security at 25 m. sig correlated with externalizing problems at 10 y. (negative), child’s obligation to obey fathers only at 10 y. (positive). |
| Lee et al. (2013), USA  FFCWS core interviews and the add-on In-Home Longitudinal Study of Pre-School Aged Children | 3024 | 36 m. | *Mother-infant*  1) Maternal warmth | 1) HOME; obs. | EC: 5 y. | *General*  1) Aggression | 1) CBCL (aggression subscale); maternal report | *Parent Initiative:* Aggression was highest in the groups characterized by low warmth at age 3. Sig negative correlation between maternal warmth at age 3 and child aggression at age 5. Sig positive correlation between maternal warmth at ages 3 and 5. |
| [Levendosky et al. (2011), USA  Mother–Infant Study](https://www.tandfonline.com/doi/full/10.1080/14616734.2020.1800769) | 150 mother-child dyads | 12 m. | *Mother-infant*  1) Attachment | 1) SSP; obs. | EC: 4 y. | *Parent-child*  1) Attachment | 1) Modified SSP; obs. | *Child Initiative:* 44.3% of children demonstrated stability of separate attachment groups with mothers from age 1 to 4 (however, p>.05). According to secure/insecure classifications 57.4% stayed concordant (p=.07). Trajectories of domestic violence (DV) and income both predicted longitudinal attachment patterns. Secure-secure and insecure-secure trajectories related to initially low DV that stayed constant or became lower and positive increase in income. |
| Lewis-Morrarty et al. (2015), USA  Longitudinal study (Fox et al., 2001) | 165 (50%) | 14 m. | *Parent-child*  1) Attachment | 1) SSP; obs. | 1) EC, MC: 4, 7 y. 2) A: 14-17 y. | *Peer*  1) Behavioral inhibition 2) social anxiety | 1) Child-peer free play (coded using POS), CCTI (shyness and sociability subscale); obs & parent-report 2) SCARED (Social Anxiety subscale); parent & self-report | *Child Initiative:* Attachment security and SCARED social anxiety not sig correlated however, the interaction of attachment and BI significantly predicted adolescent social anxiety - BI and anxiety were only associated among adolescents with histories of insecure attachment. No sig correlations between attachment and BI at 4 or 7 (both parent report and observed) |
| Licata et al. (2015), Germany  Not reported | 28 mother-child dyads | 7 m. | *Mother-infant*  1) Maternal sensitivity | 1) EAS (4th ed.); obs. | EC: 50 m. | *Parent-child*  1) Child EA | 1) EAS (4th ed.); obs. | *Parent Initiative:* Maternal sensitivity at 7 m. positively correlated with children's responsiveness, and children's involvement, at 50 m. |
| Licata et al. (2016), Germany  Not reported | 56 mother–child dyads | 7 m. | *Mother-infant*  1) EA 2) Maternal MM (appropriate & non-attuned comments) | 1) EAS (4th ed.); obs. 2) Maternal MM (Meins & Fernyhough, 2010); obs. | EC: 4 y. | *Parent-child*  1) Child EA | 1) EAS (4th ed.); obs. | *Child Initiative:* Sig. correlation between all child EA constructs at 7 m. and all child constructs at 40 m., with the exception of child involvement at 7 m. and child involvement at 50 m.. *Parent Initiative:* Sig. correlation between all maternal EA constructs at 7 m. and all child constructs at 40 m. with the exception of maternal non hostility and child involvement. |
| Liu et al. (2009), China  larger cross-cultural project (e.g., Chen et al., 1998; Liu et al., 2005) | 94 mother-child dyads (41%) | 24 m. | *Mother-infant*  1) Mother-child interactions (maternal encouragement of autonomy and connectedness) 2) Mother-child interactions (child autonomy & connectedness) | 1/2) Dyadic free play; obs | EC: 4 y. | *Peer*  1) Sociability 2) Prosocial behavior, aggression, and organization/leadership in peer interactions | 1) POS; obs. 2) Free play (coded using the social behavior coding scheme (Andison, 1999)); obs. | *Parent Initiative:* Maternal encouragement of connectedness predicted sociability and organization/leadership at 4 y. No other significant main effects of maternal behaviors were found. *Parent x Child Initiative:* Maternal encouragement of connectedness and child autonomy interaction predicted sociability (maternal encouragement of connectedness was significantly and positively associated with sociability for children with high autonomy, but not low autonomy). |
| Lorenzo et al. (2022), USA  Longitudinal study on temperament and child socioemotional development in a metropolitan Mid‐Atlantic region of the US | 291 parent‐child dyads | 36 m. | *Parent-child*  1) Parenting (task directive, dismissive, task supporting) | 1) Lab‐TAB (Goldsmith & Rothbart, 1996); obs. | MC, A: 9, 12, 15 y. | *General*  1) Social anxiety | 1) SCARED (social anxiety subscale); self-report | *Parent Initiative:* Sig. correlation between task supportive parenting at 36 m. and child social anxiety at 9 y. No other correlations significant. Interactive effect of infant temperament and parenting at 36 m. on trajectory of child social anxiety from age 9–15 (highly inhibited children engaged with high supportive and low dismissive parenting may help reduce social anxiety over time in adolescence). |
| Lounds et al., (2006), USA  longitudinal study designed to examine the development of adolescent mothers and their children (Whitman et al., 2001). | 78 adolescent mother-child dyads | 1) 12 m. 2) 6, 12, 36 m. 3) 12, 36 m. | *Mother-infant*  1) Attachment 2) Maternal responsiveness & verbal interaction 3) Child abuse potential | 1) SSP 2) Parent-child interaction (study-derived coding); obs 3) CAPI (Milner, 1986); parent-report | EC: 5 y. | *Parent-child*  1) Attachment | 1) Modified SSP; obs. | *Child Initiative:* 41% of children exhibited stable attachment classifications between 1 and 5 y. 63% children secure-secure. 42% of children insecure-secure.  *Child x Parent Initiative:* Children who were secure-secure had a higher quality of maternal interactions. For early measures of maternal interactions -responsiveness was associated with stability or change in attachment category (verbal interaction was not sig. |
| Main et al. (1985), USA  Berkeley Social Development Project (BSDP) | 40 mothers, fathers, and children (60%) | 12, 18 m. | *Parent-child*  1) Attachment | 1) SSP; obs. | EC: 6 y. | *Parent-child/Family*  1) Attachment | 1) Series of parent-child interaction episodes (Polaroid family photo; separation; separation anxiety interview; family photo presented to child; free play; parent-child reunion episode) | *Child Initiative:* Sig positive correlation between attachment security to mother at 1 and at 6 y. Sig correlation between attachment security to father at 18 m. and at 6 y. Sig positive correlation between early attachment security and later fluency of discourse for mother- and father-child dyads. Child's emotional openness at 6 was not related to security of attachment to the father but was strongly related to security attachment to the mother (positive). No relation between constructiveness of response to separation and attachment security to the father in infancy, but strong relation attachment security to mother (positive). No sig relationship between security of attachment to father and response to the family photograph at 6 y., but strong relationship for mother-child association (positive). |
| Main & Cassidy (1988), USA  Berkeley Social Development Project (BSDP) | 33 families | 12, 18 m. | *Parent-child*  1) Attachment | 1) SSP; obs. | EC: 6 y. | *Parent-child*  1) Attachment | 1) Modified SSP; obs. | *Child Initiative:* 84% of 6-year attachment categories with mother were predicted from infant attachment. 61% of 6-year attachment categories with father were predicted from infant attachment - both sig. |
| Massie et al. (2002), USA  The Brody study | 76 | 12 m. | *Mother-infant*  1) Effective mothering | 1) Parent-child interactions; obs. | AH: 30 y. | *Parent-child*  1) Attachment representations | 1) AAI; interview | *Parent Initiative:* Higher percentage of children who had experienced more favorable mothering in infancy were secure (AAI) at 30 year, compared to the less favorable mothering group (however, p>.05). |
| McGoron et al. (2012), Romania  Bucharest Early Intervention Project (BEIP) | 123 children | 30 m. | *Parent-child*  1) Caregiving quality | 1) ORCE; obs. | EC: 54 m. | *Parent-child*  1) Attachment (Indiscriminately social/disinhibited RAD; Emotionally withdrawn/Inhibited RAD) | 1) DAI (semi-structured interview); parent-report | *Parent Initiative:* Sig negative associations emerged between 30-month caregiving quality and symptoms of emotionally withdrawn/inhibited RAD. *Parent x Child Initiative:* Relationship between 30-month caregiving quality and 54-month indiscriminately social/disinhibited RAD sig mediated by 42-month attachment security. |
| Meins et al. 2017, UK  Not reported | 204 mother-child dyads | 1) 8 m. 2) 15 m. | *Mother-infant*  1) Maternal MM (appropriate and non-attuned comments) 2) Attachment | 1) Free play (coded using Ainsworth et al., 1974); obs. 2) SSP; obs. | EC: 51 m. | *Parent-child*  1) Attachment | 1) Modified SSP; obs. | *Child Initiative:* Positive weak association between four-way (secure, avoidant, resistant, disorganized/controlling) attachment from 15 to 51 m., with stability driven by secure-secure group. *Parent Initiative:* Direct effect of MM with 51 m. attachment non-sig. Sig indirect effect of nonattuned MM comments on 51 m. attachment via perspectival symbolic play (nonattuned MM comments predicted insecure attachment via their negative effect on children’s perspectival symbolic play). |
| Miller et al. (2019), USA  Family Study | 82 mothers, fathers, and infants (55%) | 1) 7 m. 2) 15 m. 3) 24 m. | *Parent-child*  1) Parental MM 2) Parental responsiveness  3) Attachment | 1) Parent-child interaction (coded using manual by Meins and Fernyhough (2015)); obs.  2) Parent-child interaction (coded using Ainsworth et al. (1971), & elicited imitation paradigm (Forman, Aksan, & Kochanska, 2004)); obs. 3) AQS; obs. | MC: 10 y. | *Parent-child*  1) Attachment | 1) IOWA; obs. | *Child Initiative:* Sig direct path from security at 2 to security at 10 y.. *Parent Initiative:* No sig direct paths from appropriate MM comments to attachment security at 10 y. No sig direct path from responsiveness to security at 10 y. Sig indirect path between responsiveness at 15 m. and at 10 y. via maternal responsiveness at 15 m. and child’s security at 2 y. (sequentially mediated). Mother’s mind-minded appropriate comments at 7 m. predicted child security at 10 y. Sig path from father responsiveness at 15 m. to child security at 10 y. Father's appropriate MM comments at 7 m. predicted child security at 10 y., and this effect was mediated by child security at 2 y.. |
| Miller-Loncar et al. (2000), USA  Not reported | 365low SES families | 24 m. | *Mother-infant*  1) Maternal behaviors (maintaining children's interests & warm sensitivity) | 1) Toy play session (naturalistic); obs. | EC: 4.5 y. | *Parent-child*  1) Child social responsiveness | 1) Parent-child interaction (including toy play); obs. | *Parent Initiative:* Sig. direct effect between maternal warm sensitivity at age 2 and child social responsiveness at 4.5 (positive). No sig direct effect of maternal maintaining and child responsiveness. |
| Mills-Koonce et al. (2022), USA  Family Life Project | 1087 | 6, 15, 24, 36 m. (composite) 36 m. | *Parent-child*  1) Parenting (sensitive and responsive; negative and controlling; cognitive stimulation) | 1) Coding behaviors from parent-child interactions (items from the HOME inventory); observational | EC: 4 y. | *General*  1) Prosocial behavior | 1) SDQ (prosocial behavior subscale); parent-report | *Parent Initiative:* Sig. positive correlation between sensitivity composite and prosocial behavior at 48 m.. |
| Mintz et al. (2011), USA  National Institute of Child Health and Human Development Study of Early Child Care and Youth Development (SECCYD) | 1,364 children (52%) | 6, 15, 24 m. (composite) 36 m. | *Mother-infant*  1) Maternal sensitivity | 1) Dyadic free play & problem-solving task; obs. | MC: 1st grade | *Child-teacher/Peer*  1) Closeness with teacher 2) Quality of interactions with peers and teachers 3) Positive interactions with peers 4) Peer competency 5) Peer status 6) Social problems 1/2/3/4/5) Relational competence (composite) 1/2) Social and relational problems (composite) | 1) STRS; teacher report 2) COS-1; observational 3) UPO; observational  4) SSRS; teacher report 5) Study-derived (Friends or Foes); self-report 6) CBCL-TRF (social problems scale); teacher report | *Parent Initiative:* Direct positive effect of maternal sensitivity on social and relational competence and problems in 1st grade. Sig direct negative effect of maternal sensitivity on social and relational problems (children whose mothers displayed lower sensitivity were more likely to display increased social and relational problems). Sig. positive correlations between maternal sensitivity and closeness with teacher, peer status. Sig. negative correlations between maternal sensitivity and conflict with teacher, negative interactions with teacher, negative interactions with peers, negative play with peers and social problems. |
| Nivison et al. (2021), USA  MLSRA | 157 | 0-24 m. | *Parent-child*  1) Adverse caregiving (abuse and neglect) | 1)Presence/absence of physical abuse, sexual abuse, neglect (coded using definitions developed by CDC); file analysis | AH: 19, 26 y. | *General attachment*  1) Attachment representations | 1) AAI; interview | *Parent Initiative:* No sig. correlation between abuse/neglect during infancy and AAI secure base script or AAI coherence. Experiences of abuse and/or neglect in infancy did not uniquely predict AAI secure base script after controlling for maternal sensitivity (composite score 3 m.-13 y.) |
| O’Connor et al. (2011), USA  NICHD Study of Early Child Care and Youth Development (SECCYD) | 1,140 mother-child dyads | 15, 36 m. | *Mother-infant*  1) Attachment | 1) Modified SSP; obs | EC: 54 m. | *Peer*  1) Positive interactions with peers  *Child-teacher* 2) Quality of relationship with teachers | 1) ORCE; obs 2) STRS; teacher report | *Child Initiative:* Sig. linear trend between attachment at 36 m. and quality of peer interactions, and quality of the teacher-child relationship at 54 m. - secure children demonstrated more positive peer interactions and higher quality teacher-child relationships than insecure-organized or disorganized/controlling children. No sig. linear trend between attachment at 15 m. and relevant outcome variables. |
| Ostrov et al. (2022), USA  Not reported | 216 caregiver-infant dyads (49%) | 7, 18, 24, 36 m. (composite) | *Parent-child*  1) Maternal harshness and sensitivity | 1) Parent-Child ERA; obs. | 1) MC: 84 m. 2) A: 13, 15 y. | *Peer*  1) Classroom aggression 2) Bullying (perpetration & victimization) | 1) BASC TRS; teacher-report 2) CBVS; self-report | *Parent Initiative:* Maternal harsh parenting positively associated with aggression. Direct negative association from harsh parenting to late adolescent bullying. Sig. indirect path from harsh parenting to early adolescent bullying via aggression. Bivariate correlations between maternal harshness and all relevant outcomes non-significant. |
| Pears et al. (2013), USA  Oregon Youth Study (OYS) | 213 father-child dyads (213 G3 offspring of 110 G2 fathers) | 21, 36 m. (composite) | *Father- child*  1) Positive parenting (composite of positive feelings about parenting & parent warmth and positive reinforcement) 2) Inconsistent discipline | 1) Pleasure in Parenting Scale (Fagot, 1995), Parent Daily Report (Chamberlain & Reid, 1987); parent-report & parent-child interaction; obs. 2) Discipline Questionnaire (Capaldi, 1995); parent-report | MC: 7 y. | *Peer*  1) Peer relations | 1) WMS (peer-preferred behaviors scale); parent- and teacher-report (composite) | *Parent Initiative: S*ig. correlation between positive parenting and peer relations (positive). Sig correlation between inconsistent parenting and peer relations (negative). |
| Priddis et al. (2009), UK  N/A | 35 mother-child dyads | 1) 15 m. 2) 24-30 m. | *Mother-infant*  1) Maternal sensitivity 2) Attachment | 1) CARE; obs. 2) Preschool SSP (PAA); obs. | EC: 6 y. | *Parent-child*  1) Attachment | 1) WAYC; interview | *Child Initiative:* No sig difference between secure and insecure ambivalent groups. Attachment continuity from 24 m. to 6 y. evident for secure, insecure avoidant and insecure ambivalent groups (significance not reported). *Parent Initiative:* Children with secure attachment representations at six y. had significantly more sensitive mothers at 15 m. of age than children with insecure avoidant representations. |
| Propper et al. (2022), USA  The Durham Child Health and Development Study | 164 families (47%) | 6 m. | *Parent-child*  1) Parenting behaviors (harsh intrusive; sensitivity) | 1) Dyadic free play task; observational | EC: 60, 84 m. (composite) | *Peer*  1) Aggression | 1) CBCL-TRF; teacher report | *Parent Initiative:* Direct effect of sensitivity on aggression problems non sig. Direct effect of harsh intrusion on aggression problems non sig. Remaining analyses not relevant. |
| Raby et al. (2013), USA  MLSRA | 143 (48%) | 12, 18 m. | *Parent-child*  1) Attachment | 1) SSP; obs. | 1) AH: 19, 26 y. 2) AH: 20-21, 26-28 y. | *General attachment*  1) Attachment representations  *Romantic* 2) Romantic attachment representations | 1) AAI; interview 2) CRI; interview | *Child Initiative:* Infant attachment security is predicted general attachment representations at age 19 (positive), but not at age 26. Infant attachment security did not sig predict romantic attachment representations at 20-21 y. or 26-28 y. |
| Raikes et al. (2008), USA  NICHD Study of Early Child Care and Youth Development (SECCYD) | 1016 | 1) 15 m. 2) 24 m. 3) 36 m. 4) 15, 24 m. (composite) | *Parent-child*  1/2/3) Attachment 2) Maternal sensitivity | 1) SSP; obs. 2) AQS; obs,  3) Modified SSP; obs. 4) Semi-structured 15-minute play periods; obs. | 1) EC, MC: 54 m., 1st grade 2) EC: 54 m. 3) MC: 1st grade | *Peer*  1) Negative attribution bias (negative attributions; aggressive solutions) 2) Social problem-solving skills 3) Loneliness and social isolation | 1) Cartoon drawings; projective 2) Social problem stories; projective 3) Modified LSDS | *Child Initiative:* Attachment security was associated with children’s peer-related representations at 54 m. and in 1st grade. Attachment security at 24 and 36 m. was associated with enhanced social problem-solving skills and less loneliness, but security of attachment at 15 m. was nonpredictive. *Parent Initiative:* Early parenting was associated with children’s peer-related representations at 54 m. and in 1st grade. Early maternal sensitivity was positively associated with later social problem-solving and negatively with aggressive responses. Early maternal sensitivity was positively associated with later social problem-solving and negatively with aggressive responses. |
| Reyes et al. (2019), Germany  Bavarian Longitudinal Study | 1181 | 1) 0-5 m. | *Parent-child*  1) Parenting | 1) PIRI & interview; parent-report | EC: 6 y. MC: 8 y. | *Peer*  1) Social inhibition 2) Friendships | 1) Interaction with stranger; obs. 2) FFI; semi structured interview | *Parent Initiative:* Good early parent–infant relationships directly predicted good inhibitory control at 20 m., which subsequently predicted low social inhibition at age 6, and higher Friendships z-scores at 8 y.. |
| Reyes et al. (2021), Germany  Bavarian Longitudinal Study | 438 (218 VP/VLBW; 220 term-born) | 1) 0-5 m. | *Parent-child*  1) Parenting | 1) PIRI & interview; parent-report | AH (trajectory): 6, 8 y. (composite), 13, 26 y. | *Peer*  1) Peer acceptance 2) Friendships 3) Peer problems | 1) PSPCSA (peer acceptance subscale), self-concept of attainment; self-report  2) FFI, number of friends; semi-structured interview 3) CBCL (peer problems subscale), SDQ (peer problems), YASR | *Parent Initiative:* Good parent-infant relationship at 5 m. predicted increased odds of being in the low peer problems trajectory. Parent-infant relationship not associated with peer acceptance or friendship trajectories. |
| Rispoli et al. (2013), USA  Early Childhood Longitudinal Study-Birth Cohort (ECLS-B) | 6850 parent-child dyads (51%) | 1) 9 m. 2) 24 m. 3) 24 m. | *Parent-child*  1) Parent responsiveness 2) Attachment 3) Parent child interactions (Child Negativity, Parent Negative Regard, Emotional Supportiveness) | 1) NCATS; obs. 2) TAS-45, AQS-revised (Waters & Deane, 1985) 3) TBT; obs. | 1) EC: 52 m. 2) EC: 64, 74 m. | *Parent-child*  1) Parent child interactions (Child Negativity)  *General* 2) Social competence | 1) TBT; obs. 2) PBKS, SSRS, ECLS-K Social Rating Scale, FACES Social Skills and Positive Approaches to Learning scale. An additional item was developed exclusively for the ECLS-B (Snow et al., 2009); parent-report | *Child Initiative:* Children rated as more negative at 2 y. were rated as more negative in preschool. Attachment Security at 2 y. was not related to Child Negativity in preschool. Child Negativity at 2 y. was not directly related to Social Competence at kindergarten entry. *Parent Initiative:* Children who experienced greater levels of Parent Responsiveness in infancy were reported as having greater Social Competence at kindergarten entry. Negativity expressed by parents at 2 y. was related to greater Child Negativity in preschool. Parent Negative Regard at 2 y. was not related to Social Competence at kindergarten entry. Sig indirect relation from 9-month Parent Responsiveness to kindergarten Social Competence through 2-year Attachment Security and preschool Parent Emotional Supportiveness. |
| Roisman et al. (2000), USA  Archival data from Sylvia Brody’s Evolution of Character study | 72 (53%) | 12 m. | *Parent-child*  1) Attachment | 1) AQS (Waters, 1994); obs. | AH: 18 y. | *Parent-child*  Attachment representations (parental idealization; parental derogation) | AAI (self-report); semi-structured interview | *Child Initiative:* Sig. negative correlational between infant attachment security and parental derogation, Non sig. correlation between infant attachment security and parental idealization. |
| Roisman et al. (2002), USA  Minnesota Mother–Child Project (Egeland & Brunquell, 1979). | 170 | 24 m. | *Mother-infant*  1) Maternal support and structure | 1) Dyadic “tool tasks” adapted from Bill Charlesworth (see Matas et al., 1978); obs. | AH; 19 y. | *General attachment*  1) Attachment representations | 1) AAI; interview | *Parent Initiative:* At 24 m., earned-secures (based on AAI discourse and mother/father loving scale) were observed to have received the best maternal support and structure of any group - significantly more so than insecures, and marginally more so than continuous-secures (p =.10). |
| Roisman et al. (2005), USA  Not reported | 73 couples (target participant - 51% female, 49% male) | 12, 18 m. (composite) | *Mother-infant*  1) Attachment | 1) SSP; obs. | AH; 20-21 y. | *Romantic*  1) Attachment | 1) CRI; semi-structured interview | *Child Initiative:* Quality of attachment in infancy was associated with attachment in young adulthood. |
| Rubin et al. (2002), Canada  N/A | 88 toddlers (46 boys and 42 girls) | 24 m. | *Mother-infant*  1) Maternal parenting behaviors (intrusiveness, derisiveness) | 1) Behavioral Inhibition Paradigm (Garcia Coll et al., 1984; Kochanska, 1991); obs. | EC: 4 y. | *Peer*  1) Withdrawn behaviors  (solitary play - solitary-passive, solitary-active, reticence) | 1) POS; obs. | *Parent Initiative:* Sig. correlation between maternal derisiveness and solitary-passive withdrawal |
| Russell et al. (2016), USA  NICHD Study of Early Child Care and Youth Development (SECCYD) | 1,264 children | 15, 36 m. | *Parent-child*  1) Attachment 2) Home environment (quality and quantity of stimulation and support available at home) 3) Maternal sensitivity | 1) SSP & Modified SSP; obs. 2) HOME; interview 3) Mother-Child Interaction ratings (Egeland & Heister, 1993); obs. | MC: 1st grade | *Peer*  1) Peer relationship satisfaction 2) Social skills 3) Classroom social competence | 1) LSDS; self-report 2) SSRS; teacher report 3) COS; obs. | *Child Initiative:* Attachment at 36 m. correlated with all 1st-grade social outcomes. *Parent Initiative:* Composite HOME (15 and 36 m.) correlated with all 1st grade social outcome; Composite maternal sensitivity (15 and 36 m.) sig correlated with all 1st grade social outcomes. HOME support and stimulation directly predictive of peer relationship satisfaction, social skills, and classroom competence. |
| Salvatore et al. (2011), USA  MLSRA; Sroufe et al., 2005 | 73 couples (target participant - 51% female, 49% male) | 12- 18 m. | *Parent-child*  1) Attachment | 1) SSP; obs. | 1/2/3) AH: 20-21 y. 4) AH: 23 y. | *Romantic*  1) Observer-rated conflict recovery 2) Emotional tone 3) Relationship satisfaction 4) Relationship stability | 1) Markman-Cox Conflict Discussion Task (Cox, 1991); obs. 2) ETI); self-report 3) Relationship Assessment Scale (Hendrick, 1988); self-report 4) Relationship status; interview | *Child Initiative:* Infant attachment security was significantly related to conflict-recovery with romantic partners 20 y. later. These children also had romantic partners who displayed better conflict recovery 20 y. later. Sig positive correlation between infant attachment (target participant) and emotional tone and relationship stability. Sig positive correlation between infant attachment (non-target partner) and conflict recovery, and relationship stability. |
| Schoenmaker et al. (2015), Netherlands  Longitudinal study (Jaffari-Bimmel et al., 2006) | 190 adopted children (47%) | 1) 12, 18, 30 m. 2) 12, 18 m. | *Parent-child*  1) Maternal sensitivity  2) Attachment | 1) Structured task; obs. 2) SSP; obs. | 1) A: 14 y. 2) AH: 23 y. | *General attachment*  1/2) Attachment representations | 1) AAI; semi-structured interview 2) ASA | *Child Initiative:* Attachment in infancy did not predict attachment representations at 14 or 23 y.. *Parent Initiative:* More maternal sensitivity in infancy predicted more secure attachment representations at 23 y.. |
| Shaw et al. (2012), USA  Pitt Mother and Child Project | 268 (100%) | 24 m. | *Mother-infant*  1) Parenting | 1) HOME (Caldwell & Bradley, 1984); obs. | MC: 10, 11, 12 y. (composite) | *General*  Callousness | APSD (Callous–Unemotional Scale) & CADS; self-report | *Parent Initiative:* HOME parenting scores at 24 m. were sig related to callousness (self-report). |
| Shi et al. (2012), Not reported  Not reported | 120 low-income adults (75%) | 18 m. | *Mother-infant*  1) Attachment  2) Disrupted mother–infant communication | 1) SSP; obs. 2) AMBIANCE (Lyons-Ruth, Bronfman, & Parsons, 1999); obs. | 1) EC, MC: 5, 7 y. 2) MC: 8 y. | *Peer*  1) Child behavior problems  *Parent-child* 2) Children’s attachment behavior toward their parents | 1) PBQ, CBCL; teacher report 2) Middle childhood disorganization and control scales; observational | *Child Initiative:* Insecure attachment at 18 m. was positively associated with children's aggressive behaviors at age 5, externalizing behaviors at age 7 and behavioral disorganization towards their mothers at 8 y.. *Parent Initiative:* Disruptive mother-infant communication, were not associated with any child outcomes. |
| Shim et al. (2021), Korea  Panel Study on Korean Children (PSKC) | 183 | 17 m. | *Mother-infant*  1) Attachment | 1) AQS-Korean version; obs. | 1) EC: 4 y. 2) EC: 6 y. | *Peer*  1) Peer play interactions 2) Behavioral problems (internalizing and externalizing) | 1) PIPPS; teach-reported survey  2) Behavior Assessment Scale based on Korean CBCL; parent-reported survey | *Child Initiative:* Secure attachment at 17 m. positively associated with children's positive interactions with children at 4 y. Secure attachment at 17 m. negatively associated with children's negative interactions with children at 4 y.. Sig indirect effect of attachment security at 17 m. on internalizing and externalizing problems at age 6 via positive peer interactions at age 4 (negative). Sig indirect effect of attachment security at 17 m. on externalizing problems at age 6 via negative peer interactions at age 4 (negative). |
| Shulman et al. (1994), USA  Egeland-Sroufe Longitudinal Sample | 32 | NR | *Parent-child*  1) Attachment | NR | MC: 11 y. | *Peer*  1) Time spent socializing with others 2) Number of friends made 3) Social network affinity (number of friends) | 1) Time spent socializing; counsellor report 2) Number of friends; self-report 3) Social network affinity; obs. & interviews | *Child Initiative:* Children with secure attachments in infancy had higher levels of social skills (counsellor report), and social network affinity at age 10, compared to children with anxious attachments. |
| Smeekens et al. (2007), Netherlands Follow up of (Van Bakel & Riksen-Walraven, 2002) | 129 | 15, 28 m. | *Parent-child*  1) Parent-child interaction 2) Attachment | 1) Parent-child interaction (coded using 7-point scale by Erickson et al., 1985); obs. 2) SSP; obs. | EC: 5 y. | *Peer*  1) Externalizing problems | 1) CBCL; teacher and parent-report | *Child Initiative:* Children's secure attachment at infancy negatively associated with children's externalizing problems at age 5.  *Parent Initiative:* Parents' negative interactions with their children at infancy positively associated with children's externalizing problems at age 5. Parents effective responses to children's behaviors, i.e., limit setting and structure, negatively associated with children's externalizing problems at age 5. |
| Steele et al. (2014), USA  NICHD Study of Early Child Care and Youth Development (SECCYD) | 673 (48%) | 15, 24, 36 m. (composite) | *Parent-child*  1/2/3) Attachment | 1) SSP; obs. 2) AQS; obs. 3) Modified SSP; obs. | AH: 18 y. | *General attachment*  1/2) Attachment | 1) ASA (adolescent version); interview 2) AAI; interview | *Child Initiative:* Proportion of times child rated as secure on early attachment measures was positively associated with secure base script knowledge (ASA) at 18 y. AAI dimensions and secure base script knowledge strongly predicted by security in infancy |
| Streit et al. (2022), USA  Fragile Families and Child Wellbeing Study (FFCWS) | 1223 | 36 m. | *Mother-infant*  1) Harsh parenting | 1) CTSPC; parent-report | A: 15 y. | *General*  1) Prosocial behavior 2) Aggression | 1) ASBI & SSRS; child-report survey 2) CBCL (aggressive behavior subscale); parent-report | *Parent Initiative:* Harsh parenting at age 3 positively associated with children's aggression at age 15. Non sig association between maternal harsh parenting and prosocial behaviors at age 15. |
| Takahashi et al. (2015), Japan  Nationwide ongoing longitudinal study in Japan (Anme & Segal, 2007; 2007) | 4 y.: 689 5 y.: 378 | 24 m. | *Parent-child*  1) Parenting practices (cognitive/emotional involvement; avoidance of restriction and punishment) | 1) Adapted 12-item tool (Anme & Segal, 2004); parent- & nurse-report | EC: 4, 5 y. | *Peer*  1) Social skills: cooperation, assertion | 1) SSQ for preschoolers; parent and teacher report | *Parent Initiative:* Parents' cognitive/emotional involvement sig associated with higher levels of children's cooperation and assertion at 5 y. but not 4 y. Parents' avoidance of punishment and restriction sig associated with higher levels of children's assertion at 5 y. but not 4 y. |
| Trapolini et al. (2007), Australia  Not reported | 92 mother-child dyads (50%) | 15 m. | *Mother-infant*  1) Attachment | 1) SSP; obs. | EC: 4 y. | *Parent-child*  1) Attachment | ASCT; projective | *Child Initiative:* No sig relationships between 15-month attachment classifications and 4-year attachment representations, all ps > 10. No sig interaction effect of 15-month attachment classification and depression (chronic vs. transient vs. never) on 4-year attachment, all ps > :10. Analyses controlled for child verbal IQ and gender. |
| van den Berg et al. (2017), Netherlands  Nijmegen Longitudinal Study on Infant and Child Development | 118 children (53%) | 15 m. | *Parent-child*  1) Parental interactive behavior (effective guidance, negative interaction) | 1) Parent-child interaction (coded using 7-point scale by Erickson et al., 1985); obs. | MC: 9 y. | *Peer*  1) Social status (preference, popularity) | 1) Study-derived; self- and peer-report | *Parent Initiative:* Sig. correlation between effective guidance and popularity (positive) in girls. No other sig correlations between predictors of interest and outcome in girls. Sig. correlation between effective guidance and preference (positive) in boys. No other sig. correlations between predictors of interest and outcome in boys. |
| van der Voort et al. (2014), Netherlands  Leiden Longitudinal Adoption Study | 160 adopted children (47%) | 12, 18, 30 m. (composite) | *Mother-infant*  1) Maternal sensitivity | 1) Structured tasks (coded using 7-point scales (Egeland et al. 1990; Erickson et al. 1985)); obs. | MC, A: 7, 14 y. | *General*  1) Withdrawn behavior 2) Behavioral inhibition | 1) CBCL; maternal-report 2) DTQ; maternal report | *Parent Initiative:* SEM - more maternal sensitivity in infancy and more maternal sensitivity 7 y. predicted less BI at 14 y. (nb. robust solution the former path non sig, p = .061). Sig direct effect from sensitivity in infancy to inhibition in 14 y. Sig indirect effect of maternal sensitivity in infancy on withdrawn behavior at 14 y. via less BI at 14 y. (negative). Sig indirect effect of BI in infancy on withdrawn behavior at 14 y. through BI at 14 and withdrawn behavior 7 y. (positive). |
| Van Ryzin et al. (2015), USA  Early Growth and Development Study (EGDS) | 361 adopted children | 27 m. | *Parent-child*  1) Emotional and verbal responsiveness | 1) HOME; interview | EC: 6 y. | *Peer*  1) Social competence | 1) SSRS; WMS (peer-preferred social behavior subscale), parent-report, teacher-report | *Parent Initiative:* Non sig associations between parent's emotional and verbal responsiveness at 27 m. and children's social competence at age 6. |
| Vieth et al. (2022), USA  MLSRA (Sroufe et al., 2005) | 158 | 12, 18 m. | *Parent-child*  1) Attachment | 1) SSP; obs. | 1) A: 12 y. 2) A: 16 y. 3) AH: 32 y. | *Peer*  1) Peer competence 2) Friendship security  3) Friendship satisfaction | 1) Study-derived, teacher-report  2/3) Friendship interview | *Child Initiative:* Sig positive association between attachment at 3 y. and children's friendship at 16 y. and adults' friendship satisfaction at 32 y.. |
| Vitaro et al. (2006), Canada  Quebec Longitudinal Study of Children’s Development | 1516 | 17 m. | *Parent-child*  1) Harsh parenting | 1) PACOTIS (Boivin et al., 2005); parent-report | EC: 6 y. | *Peer*  1) Aggression | 1) Dodge and Coie’s (1987) questionnaire; teacher/parent report survey | *Parent Initiative:* Sig association between harsh parenting at 17 m. and both proactive and reactive aggression at age 72 m. |
| Volling & Belsky (1992), USA  Longitudinal study of child and family development (Belsky, et al. 1989) | 30 | 12 m. (mothers) 13 m. (fathers) | *Parent-child*  1) Attachment | 1) SSP; obs. | EC: 6 y. | *Sibling*  1) Sibling interaction (prosocial behavior, conflict) | 1) Child-sibling interaction (study-derived coding); obs. | *Child Initiative:* Children in insecure infant-mother attachment relationships sig more likely to be in dyadic conflict with their sibling at 6 y. than children with a secure infant-mother attachment relationship. This association was not present for infant-father attachment. |
| Wang et al. (2006), China  Not reported | 181 | 24 m. | *Mother-infant*  1) Parenting strategies | 1) Study-derived; obs. | EC: 4 y. | *Peer*  1) Prosocial behaviors 2) Aggressive behaviors  3) On-task behaviors | 1/2/3) Child-peer interactions (study-derived coding adapted from CBCL); obs. | *Parent Initiative:* High-power parenting associated with lower levels of on-task behavior. Remaining correlations non sig. Sig interaction effect between child positive affect and maternal low-power parenting in predicting prosocial behavior; maternal low-power parenting positively associated with prosocial behavior for children with high positive affect scores. |
| Wartner et al. (1994), Germany  Regensburg longitudinal study | 34 | 12, 18 m. | *Mother-infant*  1) Attachment | 1) SSP; obs. | EC: 6 y. | *Parent-child*  1) Attachment | 1) Modified SSP; obs. | *Child Initiative:* Sig continuity of attachment status from infancy to 6 y.; secure/insecure attachment quality remained stable for 89.7% of the sample over time. |
| Weinfield et al. (2000), USA  Minnesota Mother–Child Project | 59 children (high developmental risk) | 12, 18 m. | *Mother-infant*  1) Attachment | 1) SSP; obs. | AH: 18-19 y. | *General attachment*  1) Attachment | 1) AAI; interview | *Child Initiative:* No sig continuity of attachment from infancy to adulthood, with many participants transitioning to insecurity. Lawful continuity - continuous attachment and discontinuous (infancy to adulthood) attachment groups sig differed on correlates of child maltreatment, maternal depression, and family functioning in early adolescence. |
| Weinfield et al. (2004), USA  Minnesota Mother-Child Project (Egeland & Brunquell, 1979 | 125 children (high developmental risk) | 12, 18 m. | *Mother-infant*  1) Attachment | 1) SSP; obs. | AH: 19 y. | *General attachment*  1) Attachment | 1) AAI; interview | *Child Initiative:* No sig continuity in attachment security from infancy to 19 y. Disorganized infants sig more likely than organized infants to be insecure or unresolved at 19 y. Sig correlates of continuity and discontinuity of attachment included infant temperament, maternal life stress, family functioning at pre-adolescence, child maltreatment and features of the home environment. |
| Zayas et al. (2011), USA  Part of an ongoing project (Mischel, Shoda, & Rodriguez, 1989). | 36 | 18 m. | *Mother-infant*  1) Maternal caregiving | 1) CARE-Index; parent-report | AH: 22 y. | *Peer*  1) Friendships  *Romantic* 2) Romantic attachment | 1/2) ECRR; self-report | *Parent Initiative:* Greater maternal sensitivity at 18 m. predicted lower avoidance to friends in adulthood. Maternal control predicted greater adult avoidance to friends. Maternal control predicted greater avoidance to partners as well as greater anxiety to partners. Interaction - maternal sensitivity predicted less avoidance and less anxiety to partners, for individuals involved in a romantic relationship. |
| Zhang (2013), China  Not reported | 115 | 33 m. | *Mother-infant*  1) Mother-child relationship, closeness & conflict | 1) CPRS; parent-report | EC: 4-5 y. | *Peer*  1) Social competence | 1) ESBRS (Social Competence subscale); parent-report | *Parent Initiative:* Mother-infant closeness at 33 m. positively associated with children's social competence at 4-5 y. Mother-infant conflict at 33 m. negatively associated with children's social competence at 4-5 y. |
| Zimmermann et al. (2001), Germany  Regensburg longitudinal study | 36 children and mothers, and 35 fathers | 12, 18 m. | *Parent-child*  1) Attachment | 1) SSP; obs. | A: 16-17 y. | *General attachment*  1) Attachment  *Peer* 2) Socially interactive behavior | 1) AAI; interview 2) Study derived; obs. | *Child Initiative:* Non sig association between mother- or father infant attachment and attachment or socially interactive behavior at 16-17 y.. |
| Ziv et al. (2004), Israel  The Haifa study of early childcare (Sagi et al., 2002) | 116 | 12 m. | *Mother-infant*  1) Attachment | 1) SSP; obs. | MC: 7-8 y. | *Peer*  1) SIP Peer Rejection (encoding, interpretation, response generation, response evaluation)  *Parent-child* 2) SIP Mother-child interaction (encoding, interpretation, response generation, response evaluation) | SIPI (Hebrew version); interview  1) Peer-group-entry scripts (study-derived coding); interview  2) Mother child interaction scripts (study-derived coding) | *Child Initiative:* Peer Rejection - Secure children sig more positive evaluation (i.e., higher beliefs in positive interpersonal and instrumental outcomes) of the competent responses compared with children classified insecure. No sig differences between secure/insecure groups in negative evaluation (i.e., inept and aggressive responses). No sig difference between secure/insecure groups on any other peer rejection SIP stages - encoding, interpretation, and response generation.  Mother-child interaction - Sig differences in interpretation of secure/insecure groups (i.e., secure children described the mother’s response to a stressful situation as sig more positive (understanding) than insecure children). No sig differences between secure/insecure groups on any other mother-child interaction SIP stages - encoding, response generation, and response evaluation. |

Note. *Measures:* AAI = Adult Attachment Interview (George et al., 1996), AMBIANCE = Atypical Maternal Behavior Instrument for Assessment and Classification (Bronfman et al., 1999), AQS = Attachment Q-sort (Waters, 1995), ASBI = Adaptive Social Behavior Inventory (Hogan et al., 1992), ASI-4 = Adolescent Symptom Inventory-4 (Gadow et al., 1997), ASCT = The Attachment Story Completion Task (Bretherton et al., 1990), Attachment Script Assessment = ASA (Waters & Waters, 2006), ATVC = Attitudes Towards Violence Scale (Funk et al., 1999), BASC-TRS = Behavior Assessment System teacher rating scale (Kamphaus & Reynolds, 2015), BSI = Brief Symptom Inventory (Derogatis, 2001), CAPI = Child Abuse Potential Inventory (Milner & Wimberley, 1986), CAI = Child Attachment Interview (Shmueli-Goetz et al., 2008), CARE Index = Child Adult Relationship Experimental Index (Crittenden, 2005), CBCL = Child Behavior Checklist (Achenbach & Edelbrock, 1991), CCTI = Colorado Child Temperament Inventory (Buss & Plomin, 2013), CIB Manual = Coding Interactive Behavior Manual (Feldman, 1998), CPRS = Child–Parent Relationship Scale (Pianta, 1992), CRI = Current Relationship Interview (Crowell & Owens, 1996), CRSQ = Children’s Rejection Sensitivity Questionnaire (Downey et al., 1998), CSI-4 = Child Symptom Inventory (Gadow & Sprafkin, 1997), CTSPC = Parent–Child Conflict Tactics Scales (Straus et al., 1998), CTS = Conflict Tactics Scales (Straus, 1979), DAI = The Disturbances of Attachment Interview (Smyke & Zeanah, 1999), DTQ = Dutch Temperament Questionnaire (Kohnstamm, 1984), EAS = Emotional Availability Scales: Infancy to Early Childhood (Biringen et al., 2000), ERA = Early Relational Assessment (Clark, 1999), ESBRS = Early School Behavior Rating Scale (Caldwell & Pianta, 1991), ETI = Emotional Tone Index (Berscheid et al., 1989), FFI = Friends and Family Interview (Steele et al., 2009), FFSFP = Face-to-Face Still-Face Paradigm (Tronick et al., 1978), HBQ = Health Behavior Questionnaire (Armstrong & Goldstein, 2003), HOME = Home Observation for Measurement of the Environment (Caldwell & Bradley, 1979), IABC = Iowa Attachment Behavioral Coding (Boldt et al., 2016), ICU = Inventory of Callous-Unemotional Traits (Frick, 2004), KSS = Kerns Security Scale (Kerns et al., 1996), LTP = Lausanne Trilogue Play (Fivaz-Depeursinge & Corboz-Warnery, 1999), LSDS = Loneliness and Social Dissatisfaction Questionnaire (Asher et al., 1984), MPI = Mannheim Parental Interview (Esser et al., 1989), NCATS = Nursing Child Assessment Teaching Scale (Sumner & Spietz, 1994), OBVQ = Olweus Bullying Questionnaire (Olweus, 2007), ODD = Oppositional Defiant Disorder, ORCE = Observational Record of the Caregiving Environment (NICHD Early Child Care Research Network, 1996), PAA = Preschool Strange Situation (Crittenden, 1992), PACOTIS = Parental Cognitions and Conduct Toward the Infant Scale (Boivin et al., 2005), PBQ = Preschool Behavior Questionnaire (Behar, 1977), PEERS = PEERS Measure (Verlinden et al., 2014), PIML = People in My Life (Cook et al., 1995), PIPS = Preschool Interpersonal Problem-Solving (Shure & Spivack, 1974), PIRI = Parent-Infant Relationship Index (Breeman et al., 2017), PKBS-2 = Preschool and Kindergarten Behavior Scales-Second Edition (Merrell, 2002), PO = Play Observation Scale (Rubin, 2001), PSPCSA = Pictorial Scale of Perceived Competence and Social Acceptance for Young Children (Harter & Pike, 1984), SAA = School Age Assessment of Attachment (Crittenden, 1997), SAS = Separation Anxiety Scale (Hock et al., 1989), SAT = Separation Anxiety Test (Klagsbrun & Bowlby, 1976), SCARED = Screen for Child Anxiety Related Emotional Disorders (Birmaher et al., 1999), SCPDP = Story Completion Procedure in Doll Play (Bretherton et al., 1990), SCIP = Sensitive and Challenging Interactive Play (Matas et al., 1978), SSP = Strange Situation Procedure (Ainsworth et al., 2015), SSRS = Social Skills Rating System (Gresham & Elliot, 1990), SSQ = Social Skills Questionnaire for Preschoolers (Takahashi et al., 2008), STRS = Student–Teacher Relationship Scale (Pianta & Nimetz, 2001), TAS-45 = Toddler Attachment Sort-45 (Bimler & Kirkland, 2002; Kirkland et al., 2004), TAT = Thematic Apperception Test (Murray, 1943), TBT = Two-Bags Task (Love et al., 2005; Owen et al., 1996), TED = Tasks of Emotional Development (Cohen & Weil, 1975), TTS = Teaching Tasks Scales (Egeland et al., 1995), UPO = Unstructured Peer Observation (Rubin et al., 2011), WAYC = Windows to Attachment in Young Children (Priddis & Howieson, 2010), WMS = Walker-McConnell Scale of Social Competence and School Adjustment (Walker et al., 1991), YASR = Young Adult Self Report (Achenbach, 1997). *Study Cohorts:* ECLS-K = Early Childhood Longitudinal Study – Kindergarten Class, FFCWS = Fragile Families and Child Wellbeing Study, MLRSA = Minnesota Longitudinal Study of Risk and Adaptation, NICHD = National Institute of Child Health and Human Development. *Abbreviations:* BI = Behavioral Inhibition, CDC = Centers for Disease Control and Prevention, EA = Emotional Availability, EO = Emotional Openness, m. = months, MAOA allele = Monoamine oxidase A genes, MM = Mind-Mindedness, MR= Mental Representations, RAD = Reactive Attachment Disorder, SIP = Social Information Processing, y. = years

**Reference List of Included Studies**

Aviezer, O., Resnick, G., Sagi, A., & Gini, M. (2016). School competence in young adolescence: Links to early attachment relationships beyond concurrent self-perceived competence and representations of relationships. International Journal of Behavioral Development, 26(5), 397-409. https://doi.org/10.1080/01650250143000328

Barglow, P., Contreras, J., Kavesh, L., & Vaughn, B. E. (1998). Developmental follow-up of 6–7 year old children of mothers employed during their infancies. Child Psychiatry and Human Development, 29, 3-20.

Bar‐Haim, Y., Sutton, D. B., Fox, N. A., & Marvin, R. S. (2003). Stability and Change of Attachment at 14, 24, and 58 Months of Age: Behavior, Representation, and Life Events. Journal of Child Psychology and Psychiatry, 41(3), 381-388. https://doi.org/10.1111/1469-7610.00622

Barker, E. D., Boivin, M., Brendgen, M., Fontaine, N., Arseneault, L., Vitaro, F., Bissonnette, C., & Tremblay, R. E. (2008). Predictive validity and early predictors of peer-victimization trajectories in preschool. Archives of general psychiatry, 65(10), 1185-1192.

Barry, R. A., & Kochanska, G. (2010). A longitudinal investigation of the affective environment in families with young children: from infancy to early school age. Emotion, 10(2), 237-249. https://doi.org/10.1037/a0018485

Becker-Stoll, F., Fremmer-Bombik, E., Wartner, U., Zimmermann, P., & Grossmann, K. E. (2008). Is attachment at ages 1, 6 and 16 related to autonomy and relatedness behavior of adolescents in interaction towards their mothers? International Journal of Behavioral Development, 32(5), 372-380. https://doi.org/10.1177/0165025408093654

Bedford, R., Wagner, N. J., Rehder, P. D., Propper, C., Willoughby, M. T., & Mills-Koonce, R. W. (2017). The role of infants' mother-directed gaze, maternal sensitivity, and emotion recognition in childhood callous unemotional behaviours. Eur Child Adolesc Psychiatry, 26(8), 947-956. https://doi.org/10.1007/s00787-017-0967-1

Beijersbergen, M. D., Juffer, F., Bakermans-Kranenburg, M. J., & van, I. M. H. (2012). Remaining or becoming secure: parental sensitive support predicts attachment continuity from infancy to adolescence in a longitudinal adoption study. Dev Psychol, 48(5), 1277-1282. https://doi.org/10.1037/a0027442

Berlin, L. J., Cassidy, J., & Belsky, J. (1995). Loneliness in young children and infant-mother attachment: A longitudinal study. Merrill-Palmer Quarterly (1982-), 91-103.

Blandon, A. Y., Calkins, S. D., & Keane, S. P. (2010). Predicting emotional and social competence during early childhood from toddler risk and maternal behavior. Dev Psychopathol, 22(1), 119-132. https://doi.org/10.1017/S0954579409990307

Blume, J., Park, S., Cox, M., & Mastergeorge, A. M. (2022). Explicating Child-Driven Patterns of Parent-Child Responsivity in Fragile Families: A Longitudinal Approach. Front Pediatr, 10, 813486. https://doi.org/10.3389/fped.2022.813486

Boldt, L. J., Goffin, K. C., & Kochanska, G. (2020). The significance of early parent-child attachment for emerging regulation: A longitudinal investigation of processes and mechanisms from toddler age to preadolescence. Dev Psychol, 56(3), 431-443. https://doi.org/10.1037/dev0000862

Boldt, L. J., Kochanska, G., Grekin, R., & Brock, R. L. (2016). Attachment in middle childhood: predictors, correlates, and implications for adaptation. Attach Hum Dev, 18(2), 115-140. https://doi.org/10.1080/14616734.2015.1120334

Borghini, A., Despars, J., Habersaat, S., Turpin, H., Monnier, M., Ansermet, F., Hohlfeld, J., & Muller-Nix, C. (2018). Attachment in Infants with Cleft Lip and/or Palate: Marginal Security and Its Changes over Time. Infant Ment Health J, 39(2), 242-253. https://doi.org/10.1002/imhj.21696

Bornstein, M. H., & Putnick, D. L. (2021). Dyadic development in the family: Stability in mother-child relationship quality from infancy to adolescence. J Fam Psychol, 35(4), 445-456. https://doi.org/10.1037/fam0000794

Bosquet, M., & Egeland, B. (2006). The development and maintenance of anxiety symptoms from infancy through adolescence in a longitudinal sample. Dev Psychopathol, 18(2), 517-550. https://doi.org/10.1017/S0954579406060275

Boutwell, B. B., Beaver, K. M., Barnes, J. C., & Vaske, J. (2012). The developmental origins of externalizing behavioral problems: parental disengagement and the role of gene-environment interplay. Psychiatry Res, 197(3), 337-344. https://doi.org/10.1016/j.psychres.2011.12.032

Burgess, K. B., Marshall, P. J., Rubin, K. H., & Fox, N. A. (2003). Infant attachment and temperament as predictors of subsequent externalizing problems and cardiac physiology. J Child Psychol Psychiatry, 44(6), 819-831. https://doi.org/10.1111/1469-7610.00167

Cao, R., Wu, M., & Liu, M. (2019). The roles of connectedness-oriented behaviours of toddlers and mothers in predicting Chinese children’s school adjustment: a longitudinal study. Early Child Development and Care, 191(9), 1481-1493. https://doi.org/10.1080/03004430.2019.1658085

Carlson, E. A. (1998). A prospective longitudinal study of attachment disorganization/disorientation. Child Development, 69(4), 1107-1128.

Carlson, E. A., Sroufe, L. A., & Egeland, B. (2004). The construction of experience: a longitudinal study of representation and behavior. Child Dev, 75(1), 66-83. https://doi.org/10.1111/j.1467-8624.2004.00654.x

Celia, M. G., Stack, D. M., & Serbin, L. A. (2018). Developmental patterns of change in mother and child emotional availability from infancy to the end of the preschool years: A four-wave longitudinal study. Infant Behav Dev, 52, 76-88. https://doi.org/10.1016/j.infbeh.2018.05.005

Chen, X., Chen, H., Wang, L., & Liu, M. (2002). Noncompliance and child-rearing attitudes as predictors of aggressive behaviour: A longitudinal study in Chinese children. International Journal of Behavioral Development, 26(3), 225-233.

Cicchetti, D., & Barnett, D. (1991). Attachment organization in maltreated preschoolers. Development and psychopathology, 3(4), 397-411.

Cyr, M., Pasalich, D. S., McMahon, R. J., & Spieker, S. J. (2014). The longitudinal link between parenting and child aggression: the moderating effect of attachment security. Child Psychiatry Hum Dev, 45(5), 555-564. https://doi.org/10.1007/s10578-013-0424-4

Dallaire, D. H., & Weinraub, M. (2005). Predicting children's separation anxiety at age 6: the contributions of infant-mother attachment security, maternal sensitivity, and maternal separation anxiety. Attach Hum Dev, 7(4), 393-408. https://doi.org/10.1080/14616730500365894

Dantchev, S., & Wolke, D. (2019). Trouble in the nest: Antecedents of sibling bullying victimization and perpetration. Dev Psychol, 55(5), 1059-1071. https://doi.org/10.1037/dev0000700

de Vries, E. E., Verlinden, M., Rijlaarsdam, J., Jaddoe, V. W. V., Verhulst, F. C., Arseneault, L., & Tiemeier, H. (2018). Like Father, like Child: Early Life Family Adversity and Children's Bullying Behaviors in Elementary School. J Abnorm Child Psychol, 46(7), 1481-1496. https://doi.org/10.1007/s10802-017-0380-8

Degnan, K. A., Hane, A. A., Henderson, H. A., Walker, O. L., Ghera, M. M., & Fox, N. A. (2015). Emergent patterns of risk for psychopathology: The influence of infant avoidance and maternal caregiving on trajectories of social reticence. Dev Psychopathol, 27(4 Pt 1), 1163-1178. https://doi.org/10.1017/S0954579415000747

Doiron, K. M., Stack, D. M., Dickson, D. J., Bouchard, S., & Serbin, L. A. (2022). Co-regulation and parenting stress over time in full-term, very low birthweight preterm, and psycho-socially at-risk infant-mother dyads: Implications for fostering the development of healthy relationships. Infant Behav Dev, 68, 101731. https://doi.org/10.1016/j.infbeh.2022.101731

Easterbrooks, M. A., Biesecker, G., & Lyons-Ruth, K. (2000). Infancy predictors of emotional availability in middle childhood: the roles of attachment security and maternal depressive symptomatology. Attach Hum Dev, 2(2), 170-187. https://doi.org/10.1080/14616730050085545

Englund, M. M., Kuo, S. I., Puig, J., & Collins, W. A. (2011). Early Roots of Adult Competence: The Significance of Close Relationships from Infancy to Early Adulthood. Int J Behav Dev, 35(6), 490-496. https://doi.org/10.1177/0165025411422994

Ensor, R., & Hughes, C. (2009). With a little help from my friends: maternal social support, via parenting, promotes willingness to share in preschoolers born to young mothers. Infant and Child Development, 19(2), 127-141. https://doi.org/10.1002/icd.643

Ettekal, I., Eiden, R. D., Nickerson, A. B., Molnar, D. S., & Schuetze, P. (2020). Developmental cascades to children's conduct problems: The role of prenatal substance use, socioeconomic adversity, maternal depression and sensitivity, and children's conscience. Dev Psychopathol, 32(1), 85-103. https://doi.org/10.1017/S095457941800144X

Feldman, R., Bamberger, E., & Kanat-Maymon, Y. (2013). Parent-specific reciprocity from infancy to adolescence shapes children's social competence and dialogical skills. Attach Hum Dev, 15(4), 407-423. https://doi.org/10.1080/14616734.2013.782650

Fihrer, I., & McMahon, C. (2009). Maternal state of mind regarding attachment, maternal depression and children's family drawings in the early school years. Attach Hum Dev, 11(6), 537-556. https://doi.org/10.1080/14616730903282498

Fish, M. (2004). Attachment in infancy and preschool in low socioeconomic status rural Appalachian children: Stability and change and relations to preschool and kindergarten competence. Development and psychopathology, 16(2), 293-312.

Galan, C. A., Choe, D. E., Forbes, E. E., & Shaw, D. S. (2017). The interaction between monoamine oxidase A and punitive discipline in the development of antisocial behavior: Mediation by maladaptive social information processing. Dev Psychopathol, 29(4), 1235-1252. https://doi.org/10.1017/S0954579416001279

Girme, Y. U., Jones, R. E., Fleck, C., Simpson, J. A., & Overall, N. C. (2021). Infants' attachment insecurity predicts attachment-relevant emotion regulation strategies in adulthood. Emotion, 21(2), 260-272. https://doi.org/10.1037/emo0000721

Gloger-Tippelt, G., Gomille, B., Koenig, L., & Vetter, J. (2002). Attachment representations in 6-year-olds: Related longitudinally to the quality of attachment in infancy and mothers' attachment representations. Attachment & Human Development, 4(3), 318-339.

Godleski, S. A., Eiden, R. D., Kachadourian, L., & Lucke, J. F. (2019). Etiological pathways to rejection sensitivity in a high-risk sample. Personality and Social Psychology Bulletin, 45(5), 715-727.

Goffin, K. C., Boldt, L. J., & Kochanska, G. (2018). A Secure Base from which to Cooperate: Security, Child and Parent Willing Stance, and Adaptive and Maladaptive Outcomes in two Longitudinal Studies. J Abnorm Child Psychol, 46(5), 1061-1075. https://doi.org/10.1007/s10802-017-0352-z

Goldberg, R. E., Tienda, M., Eilers, M., & McLanahan, S. S. (2019). Adolescent Relationship Quality: Is There an Intergenerational Link? J Marriage Fam, 81(4), 812-829. https://doi.org/10.1111/jomf.12578

Grossmann, K., Grossmann, K. E., Fremmer‐Bombik, E., Kindler, H., Scheuerer‐Englisch, H., & Zimmermann, A. P. (2002). The Uniqueness of the Child–Father Attachment Relationship: Fathers’ Sensitive and Challenging Play as a Pivotal Variable in a 16‐year Longitudinal Study. Social Development, 11(3), 301-337. https://doi.org/10.1111/1467-9507.00202

Hamilton, C. E. (2000). Continuity and discontinuity of attachment from infancy through adolescence. Child Dev, 71(3), 690-694. https://doi.org/10.1111/1467-8624.00177

Harris, M., MacMillan, H., Andrews, K., Atkinson, L., Kimber, M., England-Mason, G., & Gonzalez, A. (2021). Maternal adverse childhood experiences, executive function & emotional availability in mother-child dyads. Child Abuse Negl, 111, 104830. https://doi.org/10.1016/j.chiabu.2020.104830

Haydon, K. C., Collins, W. A., Salvatore, J. E., Simpson, J. A., & Roisman, G. I. (2012). Shared and distinctive origins and correlates of adult attachment representations: the developmental organization of romantic functioning. Child Dev, 83(5), 1689-1702. https://doi.org/10.1111/j.1467-8624.2012.01801.x

Hedenbro, M., & Rydelius, P. A. (2014). Early interaction between infants and their parents predicts social competence at the age of four. Acta paediatrica, 103(3), 268-274.

Heuser, K. M., Jaekel, J., & Wolke, D. (2018). Origins and Predictors of Friendships in 6- to 8-Year-Old Children Born at Neonatal Risk. J Pediatr, 193, 93-101 e105. https://doi.org/10.1016/j.jpeds.2017.09.072

Howes, C., & Hamilton, C. E. (1992). Children's relationships with child care teachers: Stability and concordance with parental attachments. Child Development, 63(4), 867-878.

Howes, C., Vu, J. A., & Hamilton, C. (2011). Mother-Child Attachment Representation and Relationships Over Time in Mexican-Heritage Families. Journal of Research in Childhood Education, 25(3), 228-247. https://doi.org/10.1080/02568543.2011.579863

Hubbs-Tait, L., Osofsky, J. D., Hann, D. M., & Culp, A. M. (1994). Predicting behavior problems and social competence in children of adolescent mothers. Family relations, 439-446.

Jacobsen, T., Hibbs, E., & Ziegenhain, U. (2000). Maternal expressed emotion related to attachment disorganization in early childhood: A preliminary report. Journal of Child Psychology and Psychiatry, 41(7), 899-906.

Jacobsen, T., Huss, M., Fendrich, M., Kruesi, M. J., & Ziegenhain, U. (1997). Children's ability to delay gratification: longitudinal relations to mother—child attachment. The Journal of Genetic Psychology, 158(4), 411-426.

Kim, S., & Kochanska, G. (2017). Relational antecedents and social implications of the emotion of empathy: Evidence from three studies. Emotion, 17(6), 981-992. https://doi.org/10.1037/emo0000297

Kim, S., Kochanska, G., Boldt, L. J., Nordling, J. K., & O'Bleness, J. J. (2014). Developmental trajectory from early responses to transgressions to future antisocial behavior: evidence for the role of the parent-child relationship from two longitudinal studies. Dev Psychopathol, 26(1), 93-109. https://doi.org/10.1017/S0954579413000850

Kochanska, G., Barry, R. A., Aksan, N., & Boldt, L. J. (2008). A developmental model of maternal and child contributions to disruptive conduct: the first six years. J Child Psychol Psychiatry, 49(11), 1220-1227. https://doi.org/10.1111/j.1469-7610.2008.01932.x

Kochanska, G., & Kim, S. (2012). Toward a new understanding of legacy of early attachments for future antisocial trajectories: evidence from two longitudinal studies. Dev Psychopathol, 24(3), 783-806. https://doi.org/10.1017/S0954579412000375

Kochanska, G., Kim, S., & Boldt, L. J. (2015). (Positive) power to the child: The role of children's willing stance toward parents in developmental cascades from toddler age to early preadolescence. Dev Psychopathol, 27(4 Pt 1), 987-1005. https://doi.org/10.1017/S0954579415000644

Lee, S. J., Altschul, I., & Gershoff, E. T. (2013). Does warmth moderate longitudinal associations between maternal spanking and child aggression in early childhood? Dev Psychol, 49(11), 2017-2028. https://doi.org/10.1037/a0031630

Levendosky, A. A., Bogat, G. A., Huth-Bocks, A. C., Rosenblum, K., & von Eye, A. (2011). The effects of domestic violence on the stability of attachment from infancy to preschool. Journal of Clinical Child & Adolescent Psychology, 40(3), 398-410.

Lewis-Morrarty, E., Degnan, K. A., Chronis-Tuscano, A., Pine, D. S., Henderson, H. A., & Fox, N. A. (2015). Infant attachment security and early childhood behavioral inhibition interact to predict adolescent social anxiety symptoms. Child Dev, 86(2), 598-613. https://doi.org/10.1111/cdev.12336

Licata, M., Kristen, S., & Sodian, B. (2016). Mother–child interaction as a cradle of theory of mind: The role of maternal emotional availability. Social Development, 25(1), 139-156.

Licata, M., Paulus, M., Kühn-Popp, N., Meinhardt, J., & Sodian, B. (2015). Infant frontal asymmetry predicts child emotional availability. International Journal of Behavioral Development, 39(6), 492-496. https://doi.org/10.1177/0165025415576816

Liu, M., Chen, X., Zheng, S., Chen, H., & Wang, L. (2009). Maternal Autonomy‐ and Connectedness‐oriented Parenting Behaviors as Predictors of Children's Social Behaviors in China. Social Development, 18(3), 671-689. https://doi.org/10.1111/j.1467-9507.2008.00501.x

Lorenzo, N. E., Novick, D. R., Seddio, K., Degnan, K. A., Henderson, H. A., Almas, A. N., Chronis-Tuscano, A., & Fox, N. A. (2022). Bidirectional and interactive effects of child temperament and parenting in early childhood on the trajectory of social anxiety in adolescence. Depress Anxiety, 39(3), 192-200. https://doi.org/10.1002/da.23224

Main, M., & Cassidy, J. (1988). Categories of response to reunion with the parent at age 6: Predictable from infant attachment classifications and stable over a 1-month period. Developmental psychology, 24(3), 415.

Main, M., Kaplan, N., & Cassidy, J. (1985). Security in infancy, childhood, and adulthood: A move to the level of representation. Monographs of the society for research in child development, 66-104.

Massie, H., & Szajnberg, N. (2017). The relationship between mothering in infancy, childhood experience and adult mental health. The International Journal of Psychoanalysis, 83(1), 35-55. https://doi.org/10.1516/agd4-fla0-94m9-5ptp

McGoron, L., Gleason, M. M., Smyke, A. T., Drury, S. S., Nelson, C. A., 3rd, Gregas, M. C., Fox, N. A., & Zeanah, C. H. (2012). Recovering from early deprivation: attachment mediates effects of caregiving on psychopathology. J Am Acad Child Adolesc Psychiatry, 51(7), 683-693. https://doi.org/10.1016/j.jaac.2012.05.004

McGoron, L., Gleason, M. M., Smyke, A. T., Drury, S. S., Nelson III, C. A., Gregas, M. C., Fox, N. A., & Zeanah, C. H. (2012). Recovering from early deprivation: attachment mediates effects of caregiving on psychopathology. Journal of the american academy of child & adolescent psychiatry, 51(7), 683-693.

Meins, E., Bureau, J. F., & Fernyhough, C. (2018). Mother–child attachment from infancy to the preschool years: Predicting security and stability. Child Development, 89(3), 1022-1038.

Miller, J. E., Kim, S., Boldt, L. J., Goffin, K. C., & Kochanska, G. (2019). Long-term sequelae of mothers' and fathers' mind-mindedness in infancy: A developmental path to children's attachment at age 10. Dev Psychol, 55(4), 675-686. https://doi.org/10.1037/dev0000660

Miller-Loncar, C. L., Landry, S. H., Smith, K. E., & Swank, P. R. (2000). The influence of complexity of maternal thoughts on sensitive parenting and children's social responsiveness. Journal of Applied Developmental Psychology, 21(3), 335-356.

Mills-Koonce, W. R., Towe-Goodman, N., Swingler, M. M., & Willoughby, M. T. (2022). Profiles of family-based social experiences in the first 3 years predict early cognitive, behavioral, and socioemotional competencies. Dev Psychol, 58(2), 297-310. https://doi.org/10.1037/dev0001287

Mintz, T. M., Hamre, B. K., & Hatfield, B. E. (2011). The Role of Effortful Control in Mediating the Association Between Maternal Sensitivity and Children's Social and Relational Competence and Problems in First Grade. Early Education and Development, 22(3), 360-387. https://doi.org/10.1080/10409289.2011.569317

Nivison, M. D., Facompre, C. R., Raby, K. L., Simpson, J. A., Roisman, G. I., & Waters, T. E. A. (2021). Childhood abuse and neglect are prospectively associated with scripted attachment representations in young adulthood. Dev Psychopathol, 33(4), 1143-1155. https://doi.org/10.1017/S0954579420000528

O'Connor, E., Bureau, J. F., McCartney, K., & Lyons‐Ruth, K. (2011). Risks and outcomes associated with disorganized/controlling patterns of attachment at age three years in the National Institute of Child Health & Human Development Study of Early Child Care and Youth Development. Infant Mental Health Journal, 32(4), 450-472. https://doi.org/10.1002/imhj.20305

Ostrov, J. M., Perry, K. J., Eiden, R. D., Nickerson, A. B., Schuetze, P., Godleski, S. A., & Shisler, S. (2022). Development of Bullying and Victimization: An Examination of Risk and Protective Factors in a High-Risk Sample. J Interpers Violence, 37(9-10), 5958-5984. https://doi.org/10.1177/08862605211067026

Pears, K. C., Kim, H. K., Capaldi, D., Kerr, D. C. R., & Fisher, P. A. (2013). Father-child transmission of school adjustment: a prospective intergenerational study. Dev Psychol, 49(4), 792-803. https://doi.org/10.1037/a0028543

Priddis, L. E., & Howieson, N. D. (2009). The vicissitudes of mother–infant relationships between birth and six years. Early Child Development and Care, 179(1), 43-53. https://doi.org/10.1080/03004430600912264

Propper, C. B., McLaughlin, K., Goldblum, J., Camerota, M., Gueron-Sela, N., Mills-Koonce, W. R., & Wagner, N. J. (2022). Parenting and maternal reported child sleep problems in infancy predict school-age aggression and inattention. Sleep Health, 8(1), 62-68. https://doi.org/10.1016/j.sleh.2021.11.010

Raby, K. L., Cicchetti, D., Carlson, E. A., Egeland, B., & Collins, W. A. (2013). Genetic contributions to continuity and change in attachment security: a prospective, longitudinal investigation from infancy to young adulthood. J Child Psychol Psychiatry, 54(11), 1223-1230. https://doi.org/10.1111/jcpp.12093

Raikes, H. A., & Thompson, R. A. (2008). Attachment security and parenting quality predict children's problem-solving, attributions, and loneliness with peers. Attach Hum Dev, 10(3), 319-344. https://doi.org/10.1080/14616730802113620

Reyes, L. M., Jaekel, J., Bartmann, P., & Wolke, D. (2021). Peer relationship trajectories in very preterm and term individuals from childhood to early adulthood. Journal of Developmental & Behavioral Pediatrics, 42(8), 621-630.

Reyes, L. M., Jaekel, J., Heuser, K. M., & Wolke, D. (2019). Developmental cascades of social inhibition and friendships in preterm and full‐term children. Infant and Child Development, 28(6), e2165.

Rispoli, K. M., McGoey, K. E., Koziol, N. A., & Schreiber, J. B. (2013). The relation of parenting, child temperament, and attachment security in early childhood to social competence at school entry. J Sch Psychol, 51(5), 643-658. https://doi.org/10.1016/j.jsp.2013.05.007

Roisman, G. I., Bahadur, M. A., & Oster, H. (2000). Infant attachment security as a discriminant predictor of career development in late adolescence. Journal of Adolescent Research, 15(5), 531-545.

Roisman, G. I., Collins, W. A., Sroufe, L. A., & Egeland, B. (2005). Predictors of young adults' representations of and behavior in their current romantic relationship: prospective tests of the prototype hypothesis. Attach Hum Dev, 7(2), 105-121. https://doi.org/10.1080/14616730500134928

Roisman, G. L., Padron, E., Sroufe, L. A., & Egeland, B. (2002). Earned-secure attachment status in retrospect and prospect. Child Dev, 73(4), 1204-1219. https://doi.org/10.1111/1467-8624.00467

Rubin, K. H., Burgess, K. B., & Hastings, P. D. (2002). Stability and social-behavioral consequences of toddlers' inhibited temperament and parenting behaviors. Child Dev, 73(2), 483-495. https://doi.org/10.1111/1467-8624.00419

Russell, B. S., Lee, J. O., Spieker, S., & Oxford, M. L. (2016). Parenting and Preschool Self-Regulation as Predictors of Social Emotional Competence in 1st Grade. J Res Child Educ, 30(2), 153-169. https://doi.org/10.1080/02568543.2016.1143414

Salvatore, J. E., Kuo, S. I., Steele, R. D., Simpson, J. A., & Collins, W. A. (2011). Recovering from conflict in romantic relationships: a developmental perspective. Psychol Sci, 22(3), 376-383. https://doi.org/10.1177/0956797610397055

Schoenmaker, C., Juffer, F., van, I. M. H., Linting, M., van der Voort, A., & Bakermans-Kranenburg, M. J. (2015). From maternal sensitivity in infancy to adult attachment representations: a longitudinal adoption study with secure base scripts. Attach Hum Dev, 17(3), 241-256. https://doi.org/10.1080/14616734.2015.1037315

Shaw, D. S., Hyde, L. W., & Brennan, L. M. (2012). Early predictors of boys' antisocial trajectories. Dev Psychopathol, 24(3), 871-888. https://doi.org/10.1017/S0954579412000429

Shi, Z., Bureau, J. F., Easterbrooks, M. A., Zhao, X., & Lyons-Ruth, K. (2012). Childhood Maltreatment and Prospectively Observed Quality of Early Care as Predictors of Antisocial Personality Disorder Features. Infant Ment Health J, 33(1), 55-96. https://doi.org/10.1002/imhj.20295

Shim, S. Y., & Lim, S. A. (2019). Korean infant–mother attachment security: longitudinal predictions of peer play interactions and behavioural problems in early childhood. Early Child Development and Care, 191(1), 36-48. https://doi.org/10.1080/03004430.2019.1598400

Shulman, S., Elicker, J., & Sroufe, L. A. (1994). Stages of friendship growth in preadolescence as related to attachment history. Journal of Social and Personal Relationships, 11(3), 341-361.

Smeekens, S., Riksen-Walraven, J. M., & van Bakel, H. J. (2007). Multiple determinants of externalizing behavior in 5-year-olds: a longitudinal model. J Abnorm Child Psychol, 35(3), 347-361. https://doi.org/10.1007/s10802-006-9095-y

Steele, R. D., Waters, T. E. A., Bost, K. K., Vaughn, B. E., Truitt, W., Waters, H. S., Booth-LaForce, C., & Roisman, G. I. (2014). Caregiving antecedents of secure base script knowledge: a comparative analysis of young adult attachment representations. Dev Psychol, 50(11), 2526-2538. https://doi.org/10.1037/a0037992

Streit, C., & Davis, A. N. (2022). The longitudinal links between parenting stress, harsh parenting, and adolescents’ social behaviors in Latinx families. Journal of Latinx Psychology, 10(2), 128-139. https://doi.org/10.1037/lat0000203

Takahashi, Y., Okada, K., Hoshino, T., & Anme, T. (2015). Developmental Trajectories of Social Skills during Early Childhood and Links to Parenting Practices in a Japanese Sample. PLoS One, 10(8), e0135357. https://doi.org/10.1371/journal.pone.0135357

Trapolini, T., Ungerer, J. A., & McMahon, C. A. (2010). Maternal depression and children's attachment representations during the preschool years. British Journal of Developmental Psychology, 25(2), 247-261. https://doi.org/10.1348/026151006x118739

van den Berg, Y. H., Deutz, M. H., Smeekens, S., & Cillessen, A. H. (2017). Developmental pathways to preference and popularity in middle childhood. Child Development, 88(5), 1629-1641.

van der Voort, A., Linting, M., Juffer, F., Bakermans-Kranenburg, M. J., Schoenmaker, C., & van Ijzendoorn, M. H. (2014). The development of adolescents' internalizing behavior: longitudinal effects of maternal sensitivity and child inhibition. J Youth Adolesc, 43(4), 528-540. https://doi.org/10.1007/s10964-013-9976-7

Van Ryzin, M. J., Leve, L. D., Neiderhiser, J. M., Shaw, D. S., Natsuaki, M. N., & Reiss, D. (2015). Genetic influences can protect against unresponsive parenting in the prediction of child social competence. Child Dev, 86(3), 667-680. https://doi.org/10.1111/cdev.12335

Vieth, G., Englund, M. M., & Simpson, J. A. (2022). Developmental antecedents of friendship satisfaction in adulthood. Dev Psychol, 58(12), 2401-2412. https://doi.org/10.1037/dev0001437

Vitaro, F., Barker, E. D., Boivin, M., Brendgen, M., & Tremblay, R. E. (2006). Do early difficult temperament and harsh parenting differentially predict reactive and proactive aggression? J Abnorm Child Psychol, 34(5), 685-695. https://doi.org/10.1007/s10802-006-9055-6

Volling, B. L., & Belsky, J. (1992). The contribution of mother‐child and father‐child relationships to the quality of sibling interaction: A longitudinal study. Child Development, 63(5), 1209-1222.

Wang, L., Chen, X., Chen, H., Cui, L., & Li, M. (2016). Affect and maternal parenting as predictors of adaptive and maladaptive behaviors in Chinese children. International Journal of Behavioral Development, 30(2), 158-166. https://doi.org/10.1177/0165025406063631

Wartner, U. G., Grossmann, K., Fremmer‐Bombik, E., & Suess, G. (1994). Attachment patterns at age six in south Germany: Predictability from infancy and implications for preschool behavior. Child Development, 65(4), 1014-1027.

Weinfield, N. S., Sroufe, L. A., & Egeland, B. (2000). Attachment from infancy to early adulthood in a high-risk sample: continuity, discontinuity, and their correlates. Child Dev, 71(3), 695-702. https://doi.org/10.1111/1467-8624.00178

Weinfield, N. S., Whaley, G. J., & Egeland, B. (2004). Continuity, discontinuity, and coherence in attachment from infancy to late adolescence: sequelae of organization and disorganization. Attach Hum Dev, 6(1), 73-97. https://doi.org/10.1080/14616730310001659566

Zayas, V., Mischel, W., Shoda, Y., & Aber, J. L. (2010). Roots of Adult Attachment. Social Psychological and Personality Science, 2(3), 289-297. https://doi.org/10.1177/1948550610389822

Zhang, X. (2012). Chinese Children's Relationships with Mothers During the Transition to Nursery Care: Changes and Associations with Later Growth in Social Competence. Infant Mental Health Journal, 34(1), 60-71. https://doi.org/10.1002/imhj.21354

Zimmermann, P., Maier, M. A., Winter, M., & Grossmann, K. E. (2016). Attachment and adolescents’ emotion regulation during a joint problem-solving task with a friend. International Journal of Behavioral Development, 25(4), 331-343. https://doi.org/10.1080/01650250143000157

Ziv, Y., Oppenheim, D., & Sagi-Schwartz, A. (2004). Social information processing in middle childhood: relations to infant-mother attachment. Attach Hum Dev, 6(3), 327-348. https://doi.org/10.1080/14616730412331281511

**References**

Achenbach, T. M. (1997). *Manual for the young adult self-report and young adult behavior checklist*. University of Vermont, Department of Psychiatry.

Achenbach, T. M., & Edelbrock, C. (1991). Child behavior checklist. *Burlington (vt)*, *7*, 371-392.

Ainsworth, M. D. S., Blehar, M. C., Waters, E., & Wall, S. N. (2015). *Patterns of attachment: A psychological study of the strange situation*. Psychology press.

Armstrong, J. M., & Goldstein, L. H. (2003). The MacArthur Health and Behavior Questionnaire (HBQ 1.0).

Asher, S. R., Hymel, S., & Renshaw, P. D. (1984). Loneliness in children. *Child Development*, 1456-1464.

Behar, L. B. (1977). The preschool behavior questionnaire. *Journal of Abnormal Child Psychology*, *5*, 265-275.

Berscheid, E., Snyder, M., & Omoto, A. M. (1989). The Relationship Closeness Inventory: Assessing the closeness of interpersonal relationships. *Journal of personality and Social Psychology*, *57*(5), 792.

Bimler, D., & Kirkland, J. (2002). Unifying versions and criterion sorts of the AQS with a spatial model. *The Canadian Journal of Infancy and Early Childhood*, *9*(1).

Biringen, Z., Robinson, J. L., & Emde, R. N. (2000). Appendix B: The emotional availability scales (; an abridged infancy/early childhood version). *Attachment & Human Development*, *2*(2), 256-270.

Birmaher, B., Brent, D. A., Chiappetta, L., Bridge, J., Monga, S., & Baugher, M. (1999). Psychometric properties of the Screen for Child Anxiety Related Emotional Disorders (SCARED): a replication study. *Journal of the american academy of child & adolescent psychiatry*, *38*(10), 1230-1236.

Boivin, M., Pérusse, D., Dionne, G., Saysset, V., Zoccolillo, M., Tarabulsy, G. M., Tremblay, N., & Tremblay, R. E. (2005). The genetic‐environmental etiology of parents' perceptions and self‐assessed behaviours toward their 5‐month‐old infants in a large twin and singleton sample. *Journal of Child Psychology and Psychiatry*, *46*(6), 612-630.

Boldt, L. J., Kochanska, G., Grekin, R., & Brock, R. L. (2016). Attachment in middle childhood: predictors, correlates, and implications for adaptation. *Attach Hum Dev*, *18*(2), 115-140. <https://doi.org/10.1080/14616734.2015.1120334>

Breeman, L. D., Jaekel, J., Baumann, N., Bartmann, P., & Wolke, D. (2017). Neonatal predictors of cognitive ability in adults born very preterm: a prospective cohort study. *Developmental Medicine & Child Neurology*, *59*(5), 477-483.

Bretherton, I., Ridgeway, D., & Cassidy, J. (1990). Assessing internal working models of the attachment relationship: An attachment story completion task for 3-year-olds. Portions of the material presented in this chapter were presented at the biennial meeting of the Society for Research in Child Development, Baltimore, Maryland, Apr 1987.,

Bronfman, E., Parsons, E., & Lyons-Ruth, K. (1999). Atypical Maternal Behavior Instrument for Assessment and Classification (AMBIANCE): Manual for coding disrupted affective communication. *Unpublished manuscript. Cambridge, MA: Harvard Medical School*.

Buss, A. H., & Plomin, R. (2013). The EAS approach to temperament. In *The study of temperament* (pp. 67-79). Psychology Press.

Caldwell, B. M., & Bradley, R. H. (1979). *Home observation for measurement of the environment*. University of Arkansas at Little Rock Little Rock, AR.

Caldwell, C. B., & Pianta, R. C. (1991). A measure of young children's problem and competence behaviors: The Early School Behavior Scale. *Journal of Psychoeducational Assessment*, *9*(1), 32-44.

Clark, R. (1999). The parent-child early relational assessment: A factorial validity study. *Educational and psychological measurement*, *59*(5), 821-846.

Cohen, H., & Weil, G. R. (1975). *Tasks of emotional development: A projective test for children and adolescents*. TED Associates.

Cook, E., Greenberg, M. T., & Kusche, C. (1995). People in my life: Attachment relationships in middle childhood. *Society for Research in Child Development, Indianapolis, IN*.

Crittenden, P. (1997). School-age assessment of attachment coding manual. *Unpublished manuscript, Miami, FL*.

Crittenden, P. (2005). Using the CARE-Index for screening, intervention, and research. *Online verfügbar unter:* [*http://www*](http://www)*.patcrittenden. com/images/CARE-Index. pdf, zuletzt aktualisiert am*, *3*, 2009.

Crittenden, P. M. (1992). Quality of attachment in the preschool years. *Development and psychopathology*, *4*(2), 209-241.

Crowell, J. A., & Owens, G. (1996). Current Relationship Interview and scoring system. *Unpublished manuscript, State University of New York at Stony Brook*.

Derogatis, L. R. (2001). *Brief symptom inventory 18*. Johns Hopkins University Baltimore.

Downey, G., Lebolt, A., Rincón, C., & Freitas, A. L. (1998). Rejection sensitivity and children's interpersonal difficulties. *Child Development*, *69*(4), 1074-1091.

Egeland, B., Weinfield, N., Hiester, M., Lawrence, C., Pierce, S., Chippendale, K., & Powell, J. (1995). Teaching tasks administration and scoring manual. *University of Minnesota*.

Esser, G., Blanz, B., Geisel, B., & Laucht, M. (1989). *Mannheimer Elterninterview. Strukturiertes Interview zur Erfassung von kinderpsychiatrischen Auffälligkeiten [Mannheimer Parent Interview. A structured interview for the detection of child psychiatric disorders].* Weinheim: Beltz.

Feldman, R. (1998). Coding interactive behavior manual. *Unpublished manual*.

Fivaz-Depeursinge, E., & Corboz-Warnery, A. (1999). The primary triangle. *New York*.

Frick, P. J. (2004). Inventory of callous–unemotional traits. *PLoS One*.

Funk, J. B., Elliott, R., Urman, M. L., Flores, G. T., & Mock, R. M. (1999). The attitudes towards violence scale: A measure for adolescents. *Journal of interpersonal violence*, *14*(11), 1123-1136.

Gadow, K. D., & Sprafkin, J. (1997). *Child symptom inventory 4: CSI*. Checkmate Plus Stony Brook, NY.

Gadow, K. D., Sprafkin, J., Attack, P., Phobia, S., Tics, D. M., & Anorexia, D. (1997). Adolescent symptom inventory-4 (ASI-4). *Stony Brook, NY: Checkmate Plus*.

George, C., Main, M., & Kaplan, N. (1996). Adult attachment interview. *Interpersona: An International Journal on Personal Relationships*.

Gresham, F. M., & Elliot, S. N. (1990). Social skills rating system. *PsycTESTS Dataset*.

Harter, S., & Pike, R. (1984). The pictorial scale of perceived competence and social acceptance for young children. *Child Development*, 1969-1982.

Hock, E., McBride, S., & Gnezda, M. T. (1989). Maternal separation anxiety: Mother-infant separation from the maternal perspective. *Child Development*, 793-802.

Hogan, A. E., Scott, K. G., & Bauer, C. R. (1992). The Adaptive Social Behavior Inventory (ASBI): A new assessment of social competence in high-risk three-year-olds. *Journal of Psychoeducational Assessment*, *10*(3), 230-239.

Kamphaus, R., & Reynolds, C. (2015). Behavior assessment system for children (BASC-2). *The encyclopedia of clinical psychology*, 1-6.

Kerns, K. A., Klepac, L., & Cole, A. (1996). Peer relationships and preadolescents' perceptions of security in the child-mother relationship. *Developmental psychology*, *32*(3), 457.

Kirkland, J., Bimler, D., Drawneek, A., McKim, M., & Schölmerich, A. (2004). An alternative approach for the analyses and interpretation of attachment sort items. *Early Child Development and Care*, *174*(7-8), 701-719.

Klagsbrun, M., & Bowlby, J. (1976). Responses to separation from parents: a clinical test for young children. *British Journal of Projective Psychology & Personality Study*.

Kohnstamm, G. A. (1984). Bates' Infant Characteristics Questionnaire (ICQ) in the Netherlands.

Love, J. M., Kisker, E. E., Ross, C., Constantine, J., Boller, K., Chazan-Cohen, R., Brady-Smith, C., Fuligni, A. S., Raikes, H., Brooks-Gunn, J., Tarullo, L. B., Schochet, P. Z., Paulsell, D., & Vogel, C. (2005). The effectiveness of early head start for 3-year-old children and their parents: Lessons for policy and programs [Article]. *Developmental psychology*, *41*(6), 885-901. <https://doi.org/10.1037/0012-1649.41.6.885>

Matas, L., Arend, R. A., & Sroufe, L. A. (1978). Continuity of adaptation in the second year: The relationship between quality of attachment and later competence. *Child Development*, 547-556.

Merrell, K. (2002). Preschool and kindergarten behavior scales–Second edition. *PRO-ED: Austin, TX*.

Milner, J. S., & Wimberley, R. C. (1986). Child abuse potential inventory. *Journal of Clinical Psychology*.

Murray, H. A. (1943). Thematic apperception test.

NICHD Early Child Care Research Network. (1996). Observational record of the caregiving environment. *Early Child Res Q*, *11*, 269-306.

Olweus, D. (2007). Olweus bullying questionnaire: Standard school report.

Owen, M., Barfoot, B., Vaughn, A., Domingue, G., & Ware, A. (1996). 54-month parent-child structured interaction qualitative rating scales. *NICHD Study of Early Child Care Research Consortium: Washington DC*.

Pianta, R. C. (1992). Child-parent relationship scale. *Unpublished measure, University of Virginia*, *427*.

Pianta, R. C., & Nimetz, S. (2001). Student-teacher relationship scale. *Journal of Psychoeducational Assessment*.

Priddis, L. E., & Howieson, N. D. (2010). Narrative as a window to the inner mental world of young children: attachment representations, affect and memory. *Journal of Early Childhood Research*, *8*(2), 161-174. <https://doi.org/10.1177/1476718X09345519>

Rubin, K. H. (2001). The play observation scale (POS). *Unpublished manuscript, University of Maryland*.

Rubin, K. H., Bukowski, W. M., & Laursen, B. (2011). *Handbook of peer interactions, relationships, and groups*. Guilford Press.

Shmueli-Goetz, Y., Target, M., Fonagy, P., & Datta, A. (2008). The Child Attachment Interview: a psychometric study of reliability and discriminant validity. *Developmental psychology*, *44*(4), 939.

Shure, M., & Spivack, G. (1974). Preschool interpersonal problem-solving (PIPS) test: Manual. *Philadelphia, PA: Department of Mental Health*.

Smyke, A. T., & Zeanah, C. (1999). Disturbances of attachment interview. *Unpublished manuscript*.

Steele, H., Steele, M., & Kriss, A. (2009). The friends and family interview (FFI) coding guidelines. *Unpublished manuscript*.

Straus, M. (1979). Measuring intrafamily conflict and violence: the Conflict Tactics Scales. *Journal of Marriage and the Family*, *41*, 75-88.

Straus, M. A., Hamby, S. L., Finkelhor, D., Moore, D. W., & Runyan, D. (1998). Identification of child maltreatment with the Parent-Child Conflict Tactics Scales: Development and psychometric data for a national sample of American parents. *Child abuse & neglect*, *22*(4), 249-270.

Sumner, G., & Spietz, A. (1994). *Ncast caregiver/parent-child interaction teaching manual.*

Takahashi, Y., Okada, K., Hoshino, T., & Anme, T. (2008). Social skills of preschoolers: stability of factor structures and predictive validity from a nationwide cohort study in Japan. *Japanese Journal of Educational Psychology*.

Tronick, E., Als, H., Adamson, L., Wise, S., & Brazelton, T. B. (1978). The infant's response to entrapment between contradictory messages in face-to-face interaction. *Journal of the American Academy of Child psychiatry*, *17*(1), 1-13.

Verlinden, M., Veenstra, R., Ringoot, A. P., Jansen, P. W., Raat, H., Hofman, A., Jaddoe, V. W., Verhulst, F. C., & Tiemeier, H. (2014). Detecting bullying in early elementary school with a computerized peer-nomination instrument. *Psychological assessment*, *26*(2), 628.

Walker, H. M., Mcconnell, S. R., & Lewis, T. (1991). The Walker-Mcconnell scale of social competence and school adjustment: A social skills rating scale for teachers. *Behavioral Disorders*, *16*(2), 159-160.

Waters, E. (1995). Appendix A: The attachment Q-set (version 3.0). *Monographs of the society for research in child development*, 234-246.

Waters, H. S., & Waters, E. (2006). The attachment working models concept: Among other things, we build script-like representations of secure base experiences. *Attachment & Human Development*, *8*(3), 185-197.
